# Single-mitochondrion sequencing uncovers distinct mutational patterns and heteroplasmy landscape in mouse astrocytes and neurons

**DOI:** 10.1186/s12915-024-01953-7

**Published:** 2024-07-29

**Authors:** Parnika S. Kadam, Zijian Yang, Youtao Lu, Hua Zhu, Yasemin Atiyas, Nishal Shah, Stephen Fisher, Erik Nordgren, Junhyong Kim, David Issadore, James Eberwine

**Affiliations:** 1 Department of Pharmacology, Perelman School of Medicine, University of Pennsylvania, Philadelphia, PA 19104 USA; 2 Department of Bioengineering, School of Engineering and Applied Science, University of Pennsylvania, Philadelphia, PA 19104 USA; 3 Department of Biology, School of Arts and Sciences, University of Pennsylvania, Philadelphia, PA 19104 USA

**Keywords:** Single mitochondrion, Single-nucleotide variants, Neurons, Astrocytes, Heteroplasmy

## Abstract

**Background:**

Mitochondrial (mt) heteroplasmy can cause adverse biological consequences when deleterious mtDNA mutations accumulate disrupting “normal” mt-driven processes and cellular functions. To investigate the heteroplasmy of such mtDNA changes, we developed a moderate throughput mt isolation procedure to quantify the mt single-nucleotide variant (SNV) landscape in individual mouse neurons and astrocytes. In this study, we amplified mt-genomes from 1645 single mitochondria isolated from mouse single astrocytes and neurons to (1) determine the distribution and proportion of mt-SNVs as well as mutation pattern in specific target regions across the mt-genome, (2) assess differences in mtDNA SNVs between neurons and astrocytes, and (3) study co-segregation of variants in the mouse mtDNA.

**Results:**

**(**1) The data show that specific sites of the mt-genome are permissive to SNV presentation while others appear to be under stringent purifying selection. Nested hierarchical analysis at the levels of mitochondrion, cell, and mouse reveals distinct patterns of inter- and intra-cellular variation for mt-SNVs at different sites. (2) Further, differences in the SNV incidence were observed between mouse neurons and astrocytes for two mt-SNV 9027:G > A and 9419:C > T showing variation in the mutational propensity between these cell types. Purifying selection was observed in neurons as shown by the Ka/Ks statistic, suggesting that neurons are under stronger evolutionary constraint as compared to astrocytes. (3) Intriguingly, these data show strong linkage between the SNV sites at nucleotide positions 9027 and 9461.

**Conclusions:**

This study suggests that segregation as well as clonal expansion of mt-SNVs is specific to individual genomic loci, which is important foundational data in understanding of heteroplasmy and disease thresholds for mutation of pathogenic variants.

**Supplementary Information:**

The online version contains supplementary material available at 10.1186/s12915-024-01953-7.

## Background

Mitochondria are subcellular organelles that govern cellular processes including bioenergetics, calcium buffering, DNA repair, cell cycle, cell death, and organelle signal transduction [1]. In addition to mitochondrial (mt) diseases [2], these mt-driven cellular processes are dysregulated in Parkinson’s [3], Alzheimer’s [4], cardiovascular and renal diseases [5, 6], and cancer [7]. There can be as many as thousands of mitochondria in a cell depending on the cell type and metabolic state, and each mt can have up to tens of copies of circular, self-replicating mtDNA [8]. The circular mtDNA in mice and humans is about 16.3 and 16.5 kb respectively and encodes 13 key proteins involved in the electron transport chain, and two rRNAs (namely 12S and 16S rRNA interspersed by 22 tRNAs [9, 10]. Compared with the nuclear genome, mtDNA are at least 10 times more mutation prone [11], and both germline and somatic mutations can contribute to the coexistence of the wild-type and mutant alleles in the same organelle or the same cell, a phenomenon known as “heteroplasmy” [12]. Pathogenic mt heteroplasmy is implicated in maladies including myoclonus epilepsy with ragged-red fibers [2] and Huntington’s disease [13], while pervasive mt heteroplasmy was also observed in cells of healthy humans and mice [11, 14–16].

It is known that mtDNA mutations accumulate over time and can reach a “disease-threshold” causing deleterious biological consequences. One approach to understanding this process is to study and decipher the patterns of SNV distribution and accumulation enriched by selective replication [17]. To better understand the mechanistic aspects of such changes, the systematic structure of the mt heteroplasmy should be explored in context of its nested hierarchy, i.e., the level of single mitochondria, single cells, and single organisms. Key questions such as heteroplasmy across different levels, the segregation mode of heteroplasmic alleles, and the level(s) of regulation upon which selection forces may exert, have been only partially addressed. Matthews et al. [18] showed that mitochondria segregated in heteroplasmic units in human fibroblasts, while studies by Cavelier et al. [19] reported predominantly homoplasmic nucleoids in human fibroblasts. Due to technical limitations, these early pioneering studies only queried single sequence loci in mtDNA. Evidence for tissue-specific selection has been observed in mice [20] and more recently in humans [21], but these studies focused at the tissue level, leaving the question unanswered as to which cellular or sub-cellular “regulatory level” selection may act upon. In 2017, we applied single-mt sequencing to primary cultures of mouse and human neurons and astrocytes and found allelic variation within a single mitochondrion over the multiple genomes encapsulated in a single organelle. We showed larger inter- and intra-cellular single mt allele frequency (AF) variation in mouse than in human neurons, suggesting that the two species may have evolved different ways for mt segregation [22]. A limitation of this work was the small sample size for individual mitochondrion due to manual organelle collection, which limited the ability to estimate the intra-cellular mt variation.

**Fig. 1:**
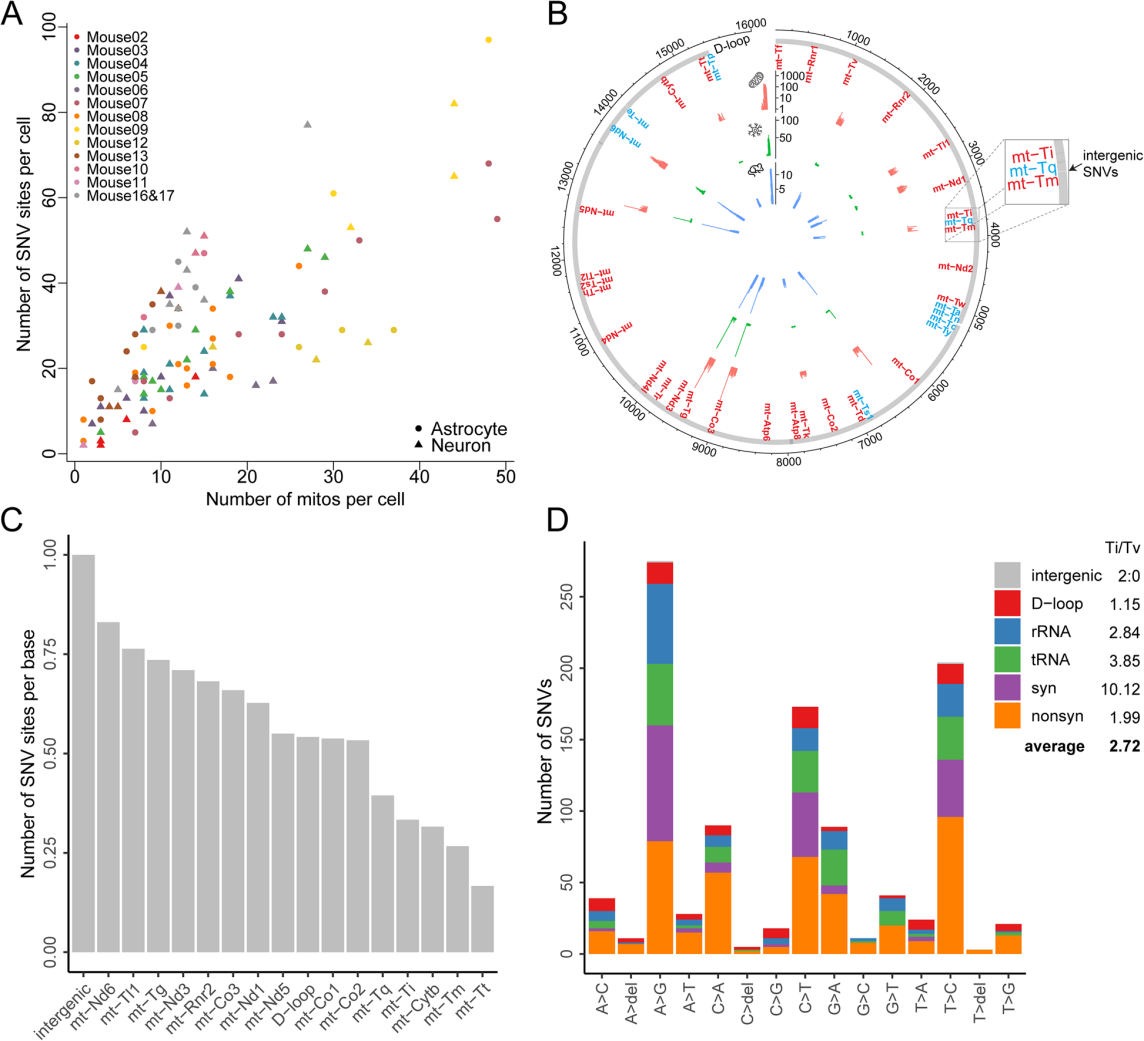
SNV distribution in SMITO dataset. **A** The number of SNVs sites per cell as a function of the number of mitochondria in each cell. Mitochondria collected from each mouse are color coded, from astrocytes and neurons are depicted by circles and triangles respectively. **B** Circos plot showing distribution and site of detected SNVs along the mitochondrial (mt)-genome. The bar height indicates the number of mitochondria (red), cells (green) and mice (blue). Thirty-seven mt genes are marked along the thick circumference (red denotes H-strand genes, blue indicates L-strand genes). **C** The number of SNVs per gene/region normalized to SMITO-analyzed target region length (bp). **D** A SNV mutational spectrum with the number of SNVs on the y-axis grouped by base substitutions: transitions (Ti) and transversions (Tv) on the *x*-axis. Ti and Tv are color-coded by region (D-loop, rRNA, tRNA) or functional impact in protein-coding region (synonymous, nonsynonymous). Analyses were performed on data from *n* = 13 mouse pups, *n* = 102 cells, and *n* = 1645 single mitochondria

To generate the single mt landscape of single-nucleotide variants (SNVs) in specific regions from dispersed cell cultures of mouse cortical brain region astrocytes and neurons, we developed a highly scalable moderate-throughput single-cell-single-mitochondria isolation procedure using fluorescence-activated mt sorting of mitochondria-associated TOMM20 antibody conjugated microbeads [23, 24]. Our approach has allowed us to study inter- as well as intra- cellular mtDNA variation from isolating an average of 10 times more single mitochondria from single neurons and astrocytes than previously reported [22]. Briefly, mouse neurons and astrocytes from brain cortex dissection were seeded on coverslips and maintained in primary neuronal cell culture at 37 °C, 5% CO_2_. The coverslips were stained with MitoTracker Red before the single neurons and astrocytes were harvested using micropipette and transferred into an Eppendorf tube. The single cells lysates were incubated with anti-TOMM20 conjugated microbeads for flow cytometry of captured mitochondria from single cells (see “Methods” for details and Fig. 1A and Additional file 1: Figure S1A for number of mitochondria isolated per cell and per mouse pup). After sorting single mitochondria into a 96-well plate, we developed a two-prong approach to amplify mt-genomes from individual single isolated mitochondria. We adapted Rolling Circle Amplification (RCA, linear amplification method) to amplify individual mt-genomes from single mitochondria producing yields large enough to perform multiple subsequent PCRs. The mitochondria ID barcoded PCR primers were designed to amplify 12 target regions containing previously reported SNVs [22] (Table 1). Using this method, we were able to detect a spectrum of known and novel SNVs from over 1645 single mitochondria from ~ 100 single astrocytes and neurons isolated from 13 mice in our single-mitochondria (SMITO) dataset. We identified 1032 SNVs showing prevalence bias based on the target regions to which they belong. Both somatic and inherited mt variants are associated with diseases. Hence, these were further classified into inherited or somatic SNVs to elucidate the influence of evolutionary processes on heteroplasmy dynamics. Comparison of these two classes of SNVs showed distinct regional differences in the occurrence and transition-transversion bias. For the inherited SNVs, AF variation at the levels of single mitochondria, cell, and animal revealed overall variation to be the largest at the animal level. Also, specific SNV sites exhibited relatively higher inter- and intra-cellular variation as compared to the variation at the animal level. Apart from the global role of mitochondria in a cell, it is known that mitochondrial metabolism in neurons vs astrocytes is different having evolved to meet specialized roles of neurons and astrocytes [25, 26]. We found that astrocytes exhibit more SNVs than neurons and two SNVs with differential incidence, suggesting distinct mutational propensity in these two cell types and purifying selection in neurons. In addition, we detected linked SNV pairs within the analyzed regions that suggest co-segregation.

**Table 1:** List of SNV target regions

SNV target region	SNV position	Gene	Reference	Variants	Target loci		Fragment size	Distance 5′ end	Distance 3′ end
1	9461	Mt-Nd3	T	C	9350	9534	185	111	73
2	12913	Mt-Nd5	C	A	12783	12973	191	130	60
3	9027	Mt-Co3	G	A	8888	9093	206	139	66
4	6543	Mt-Co1	G	A	6409	6580	172	134	37
5	3816	Mt-Tq	T	C	3750	3892	143	66	76
6	13776	Mt-Nd6	C	T	13701	13889	189	75	113
7	3079	Mt-Nd1	G	T	2970	3156	187	109	77
8	16029	D-Loop	A	G	16008	16184	177	21	155
9	7612	Mt-Co2	T	C	7540	7725	186	72	113
10	2651	Mt-Rnr2	T	C	2591	2792	202	60	141
11	15191	Mt-Cytb	C	T	15155	15330	176	36	139
12	1317	Mt-Rnr2	T	A	1201	1331	131	116	33

## Results

### SMITO SNV detection, distribution, and quality assessment in each mitochondrion sample

After QC filtering of data collected from 1645 mitochondria, 102 cells and 13 mice (the co-cultured Mouse16&17 regarded as one animal; the pup brains in such cases are trypsinized together and then plated to give rise to these mixed pup cultures that were then maintained in culture for days) (Additional file 1: Figure S1, Additional file 2: Dataset S1), 1032 SNVs distributed over 838 unique sites were obtained across 12 target regions (Table 1 lists the target regions as well as a previously reported SNV within each region); 1519 mitochondria showed at least one SNV. Out of the 12 SNVs specifically targeted from previously reported SNVs [22], we found 4 in our new samples: 9027:G > A (in 1308 mitochondria, 100 cells, 13 mice), 9461:T > C (in 1198 mitochondria, 100 cells, 13 mice), 15191:C > T (in two mitochondria, two cells, two mice), and 3816:T > C (in only one mitochondrion). We detected 4 of the 12 SNVs that were reported in Morris et al. (2017) potentially due to differences in mouse population as well as neuronal cell populations (hippocampal cells were also used in the previous study as opposed to only cortical neuronal cultures used for the SMITO study). Among previously reported SNVs [22] (*n* = 285), 35 are within the sequencing interval of SMITO and 22/35 (63%) SNVs were observed by our SMITO data. On average, we detected 4.5 variant sites within the targeted regions (~ 1/10th of genome) per mitochondrion (Additional file 3: Figure S2) and the number of variant sites per cell scales linearly with the mitochondria count in a single cell (Fig. 1A). The SNV count was found to be significantly associated with the mouse identity (ANOVA *p* < 2e − 16), suggesting that mice can have different amounts of mutation load, likely through differential inheritance and total somatic mutations.

Figure 1B shows the distribution of SNVs with respect to mt-genes and the number of mitochondria, cells, and mice sharing each variant site. After our filtering criteria (see “Methods”), most (75%) of the SNV positions were found to be shared by at most three mitochondria (three cells or two mice). The Region 8 in the D-loop (16008–16184) showed most abundant SNVs. The observed spikes at SNVs 6430, 6432, 9027, 9461, and 12831 indicate highly ubiquitous SNVs. Single-mt-SNVs at 6430 and 6432 were prevalent in a subset of 5 mice. The width of the shaded patches in Fig. 1B indicate dispersion of the SNVs within a specific target region like in region 1 (9350–9534, mt-ND3), 3 (8888–9093, mt-Co3), 6 (13701–13889, mt-Nd6), and 7 (2970–3156, mt-Nd1). The intergenic target region contained the highest number of SNV sites per base (= 1 as a result of 2 SNVs for 2 bases coverage), followed by the region in mt-Nd6 (98 sites for 118 bases) as shown in Fig. 1C.

We further analyzed the spectrum of mt mutations. Among the 1032 unique SNVs in total (Fig. 1D), transitions (A <  > G, T <  > C) predominated base substitutions (Figure S3), consistent with findings in mouse brain and muscle [16]. Our analysis reveals that the occurrences of C > T and G > A changes were as frequent as T > C and A > G changes (Additional file 4: Figure S3) indicating that deamination errors [27] were unlikely to affect our dataset. There are 440 nonsynonymous, 189 synonymous, 161 tRNA, 147 rRNA, and 93 D-loop as well as two intergenic (between mt-Tq and mt-Tm) SNVs (Fig. 1D). Among these, 42 are predicted to be high impact (generating stop codon or reading frame shifted) (Table 2) and Additional file 5: Dataset S2 lists the potential functional impact of these 1032 SNVs along with SIFT (Sorting Intolerant from Tolerant) [28–30] prediction (whether an amino acid replacement has an impact on protein functionality) results. Using the SIFT prediction for non-synonymous SNVs, we tested if greater number of mitochondria and cells shared “tolerated” SNVs as compared to the “deleterious” SNVs using Wilcoxon’s rank-sum test (Additional file 6: Figure S4). However, the effect size was minor and no statistical significance was detected (including Poisson regression test).

**Table 2:** List of the high-impact SNVs annotated by Variant Effect Predictor

SNV	Number of mice	Number of cells	Number of Mt	Gene	Effect	CDS position	Protein position	Amino acids	Codons
12831:A > T	13	52	107	mt-Nd5	stop_gained	1090	364	K/*	Aaa/Taa
13766:A > del	9	22	29	mt-Nd6	frameshift	305	102	L/X	tTa/ta
13796:A > del	4	5	5	mt-Nd6	frameshift	275	92	L/X	tTa/ta
9459:A > G	3	6	6	mt-Nd3	start_lost	1	1	M/V	Att/Gtt
13788:C > T	3	6	6	mt-Nd6	stop_gained	283	95	G/*	Ggg/Agg
3106:C > A	2	2	2	mt-Nd1	stop_gained	356	119	S/*	tCa/tAa
6472:C > A	2	2	3	mt-Co1	stop_gained	1145	382	S/*	tCa/tAa
7595:C > T	2	2	2	mt-Co2	stop_gained	583	195	Q/*	Caa/Taa
12814:A > G	2	2	2	mt-Nd5	stop_gained	1073	358	K/*	aAa/aGa
13747:A > T	2	3	3	mt-Nd6	stop_gained	324	108	Y/*	taT/taA
13785:C > del	2	2	2	mt-Nd6	frameshift	286	96	V/X	Gtg/tg
13805:A > del	2	2	3	mt-Nd6	frameshift	266	89	L/X	tTg/tg
13814:C > T	2	3	3	mt-Nd6	stop_gained	257	86	W/*	tGa/tAa
3007:G > A	1	1	1	mt-Nd1	stop_gained	257	86	W/*	tGa/tAa
3034:T > A	1	1	1	mt-Nd1	stop_gained	284	95	L/*	tTa/tAa
3103:G > A	1	1	1	mt-Nd1	stop_gained	353	118	W/*	tGa/tAa
6430:A > del	1	1	1	mt-Co1	frameshift	1103	368	H/X	cAc/cc
6455:T > del	1	1	1	mt-Co1	frameshift	1128	376	H/X	caT/ca
6477:G > A	1	1	1	mt-Co1	stop_gained	1150	384	G/*	Gga/Aga
6514:G > A	1	1	1	mt-Co1	stop_gained	1187	396	W/*	tGa/tAa
6529:C > A	1	1	1	mt-Co1	stop_gained	1202	401	S/*	tCa/tAa
6534:T > del	1	1	1	mt-Co1	frameshift	1207	403	F/X	Ttc/tc
7560:C > G	1	1	1	mt-Co2	stop_gained	548	183	T/*	aCa/aGa
7561:A > del	1	1	1	mt-Co2	frameshift	549	183	T/X	acA/ac
7591:T > G	1	1	1	mt-Co2	stop_gained	579	193	Y/*	taT/taG
7646:G > T	1	1	1	mt-Co2	stop_gained	634	212	E/*	Gaa/Taa
7660:A > del	1	1	1	mt-Co2	frameshift	648	216	L/X	ctA/ct
7670:G > T	1	1	1	mt-Co2	stop_gained	658	220	E/*	Gaa/Taa
8964:G > A	1	1	1	mt-Co3	stop_gained	358	120	G/*	Gga/Aga
8993:C > del	1	1	2	mt-Co3	frameshift	387	129	V/X	gtC/gt
9377:T > G	1	1	1	mt-Co3	stop_gained	771	257	Y/*	taT/taG
9384:G > A	1	1	1	mt-Co3	stop_gained	778	260	G/*	Gga/Aga
12867:G > A	1	1	1	mt-Nd5	stop_gained	1126	376	G/*	Gga/Aga
12885:G > A	1	1	1	mt-Nd5	stop_gained	1144	382	G/*	Gga/Aga
13734:C > T	1	1	1	mt-Nd6	stop_gained	337	113	G/*	Gga/Aga
13744:A > T	1	1	1	mt-Nd6	stop_gained	327	109	Y/*	taT/taA
13764:T > del	1	1	1	mt-Nd6	frameshift	307	103	I/X	Att/tt
13824:C > T	1	1	1	mt-Nd6	stop_gained	247	83	G/*	Gga/Aga
13826:C > T	1	2	2	mt-Nd6	stop_gained	245	82	W/*	tGg/tAg
13827:A > T	1	1	2	mt-Nd6	stop_gained	244	82	W/*	Tgg/Agg
13833:C > A	1	2	2	mt-Nd6	stop_gained	238	80	E/*	Gag/Tag
15275:A > del	1	1	1	mt-Cytb	frameshift	1131	377	L/X	ctA/ct

We also checked SNV impact on tRNAs, focusing on hits in the anticodon region. In total, there were 41 SNVs (at 40 unique sites) affecting tRNAs; among them, 24 hit mt-Tg, 12 hit mt-Tl1, 3 hit mt-Tm and 2 hit mt-Tq. By assessing distance from SNV to anticodon center, a single candidate, 9423:C > T was identified, which would impact the 3rd position of the anticodon TCC > TCT (Additional file 7: Figure S5). The mutated tRNA may still be able to recognize the codon GGA because U can form a wobble pair with G. Since the vertebrate mitochondrial stop codon is AGR (R = A/G), we reasoned that 9423:C > T might rescue premature termination caused by nonsense mutations (e.g., 6472:C > A, 12831:A > T). Yet, the low frequency of 9423:C > T (observed in only 4 mitochondria from 4 cells in 3 mice) hindered further association testing.

### Mouse strain associated segregating sites and inherited-somatic SNV classification

**Fig. 2:**
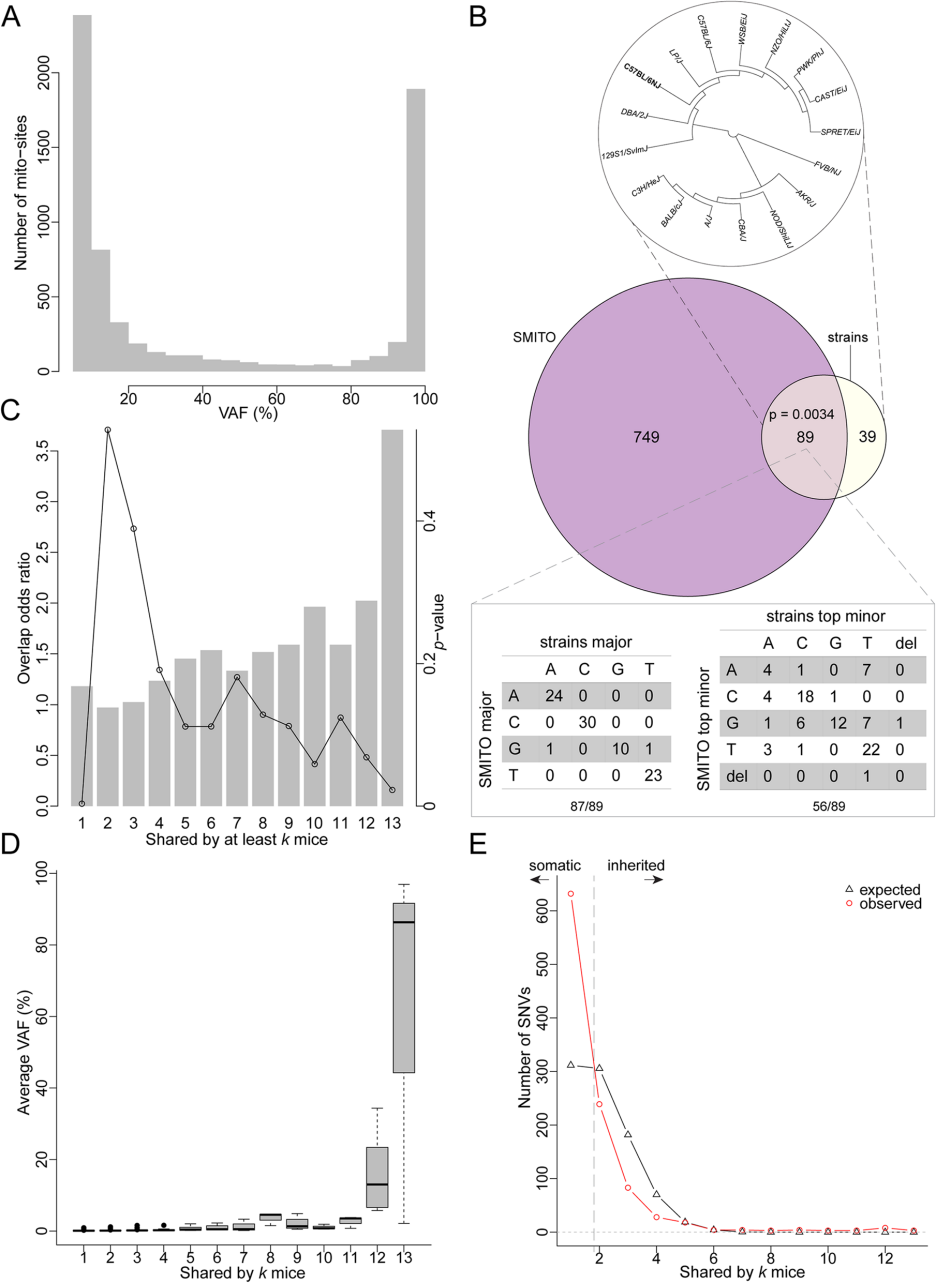
Mouse strain associated segregating sites and inherited-somatic SNV classification. **A** Distribution of the variant allele frequencies (VAFs) on the *x*-axis for all the variants/SNVs observed in all the single mitochondria samples on the *y*-axis. **B** Top: the neighbor joining tree built on 17 mouse strains’ segregating sites; middle: the Venn diagram showing the overlap between SMITO and the mouse strains data limited to the 2145 bp analyzed in this study; bottom: consistency in the major alleles (left) and top minor alleles (right) between SMITO and the mouse strains data. **C** The barplot shows the strength of the overlap of the mouse strains and SMITO data conditioned on the number of mice shared. The dotplot shows the *p*-value from hypergeometric tests. **D** The relationship between average AFs on the *y*-axis and the number of mice shared on the *x*-axis. **E** Classification of somatic and inherited SNVs by the theoretical (black triangles) and the observed (red circles) distribution of the SNV count (*y*-axis) as a function of the number of mice shared (*x*-axis). Analyses were performed on data from *n* = 13 mouse pups, *n* = 102 cells, and *n* = 1645 single mitochondria

Since each mitochondrion has multiple genomes, the frequency of each base-pair variant was estimated by read frequency and adapting the population genetics terminology, called “allele frequency” (AF). The variant AF (VAF) distribution for all SNV sites showed a U-shape with the two modes in the range of 5–10% and 95–100% (Fig. 2A), reflecting potentially neutral drift of majority of the allelic variants with 0 and 1 absorbing states. We also compared SNV sites (838) from our SMITO dataset against the segregating sites from the mitochondria of 17 mouse strains and observed a significant (hypergeometric test *p* = 0.0034) overlap (*n* = 89) (Fig. 2B). Assuming the 17 strains were an unbiased sample, the AF averaged over 17 strains was calculated and the highest AF was selected as the major allele (Additional file 8: Dataset S3) and the second highest AF as the top minor allele. Among the 89 overlapping sites, we observed a 98% match for major alleles, consistent with segregating variation seen in the strains and a 63% match for minor alleles. In theory, each site can have up to 3 minor alleles. For example, a major allele “A” can have C,G,T minor alleles or a deletion and the expected match should be merely 25% (1/4) by chance.

Given the lower likelihood of somatic mutations being shared between animals, we hypothesized that a higher prevalence of an SNV shared among animals increases the likelihood of it being inherited. Of our 838 unique SMITO sites, the non-overlapping 749 sites were most likely not derived from germline inheritance. To assess the overlap of SNVs from the mouse strain data with our SMITO dataset, we calculated the overlap odds ratio as a function of the number of mice sharing SNVs. Using the mouse strains data as a plausible pool of inherited SNVs, we observed stronger overlap when thresholding the SNVs by higher number of mice that share the SNV (Fig. 2C) as seen by the bars that depict the odds ratio and the *p*-value depicted by the dotplot within the same graph. According to the “drifting model” of neutral alleles, the “older” an SNV, the more extreme AF (i.e., towards 0 or 1). As anticipated, we observed increasingly larger AF variation when the number of mice shared went higher (Fig. 2D). We also noticed a monotonic increase in the average AF especially as the number of mice sharing SNVs exceeded 9. This trend was also true for the number of cells or mitochondria sharing SNVs (Additional file 9: Figure S6A-B). The three SNVs found to be shared by all 13 mice were 9027:G > A (average AF 86.3%), 9461:T > C (average AF 96.9%) and 12831:A > T (average AF 2.2%). Less shared SNVs are presumably “young” and are expected to have lower frequency as compared to more highly shared SNVs.

Using the hypothesis that shared SNVs among mice likely indicate inherited rather than de novo mutations, we categorized SNVs as somatic or inherited. The number of SNVs as a function of the number of mice that shared the variant was modelled (Fig. 2E). A null distribution was generated where SNVs were assumed random while SNVs per mouse were maintained. We found several SNVs unique to an individual mouse but a lower-than-expected SNVs shared by 2–4 mice (Fig. 2E). From this analysis, the SNVs unique to each mouse were designated as “somatic,” and the SNVs shared by ≥ 3 mice as “inherited” for the rigorous analysis. As a result, we classified 632 SNVs as somatic and 161 as inherited SNVs.

### Mutational preference comparison between inherited and somatic SNVs

**Fig. 3:**
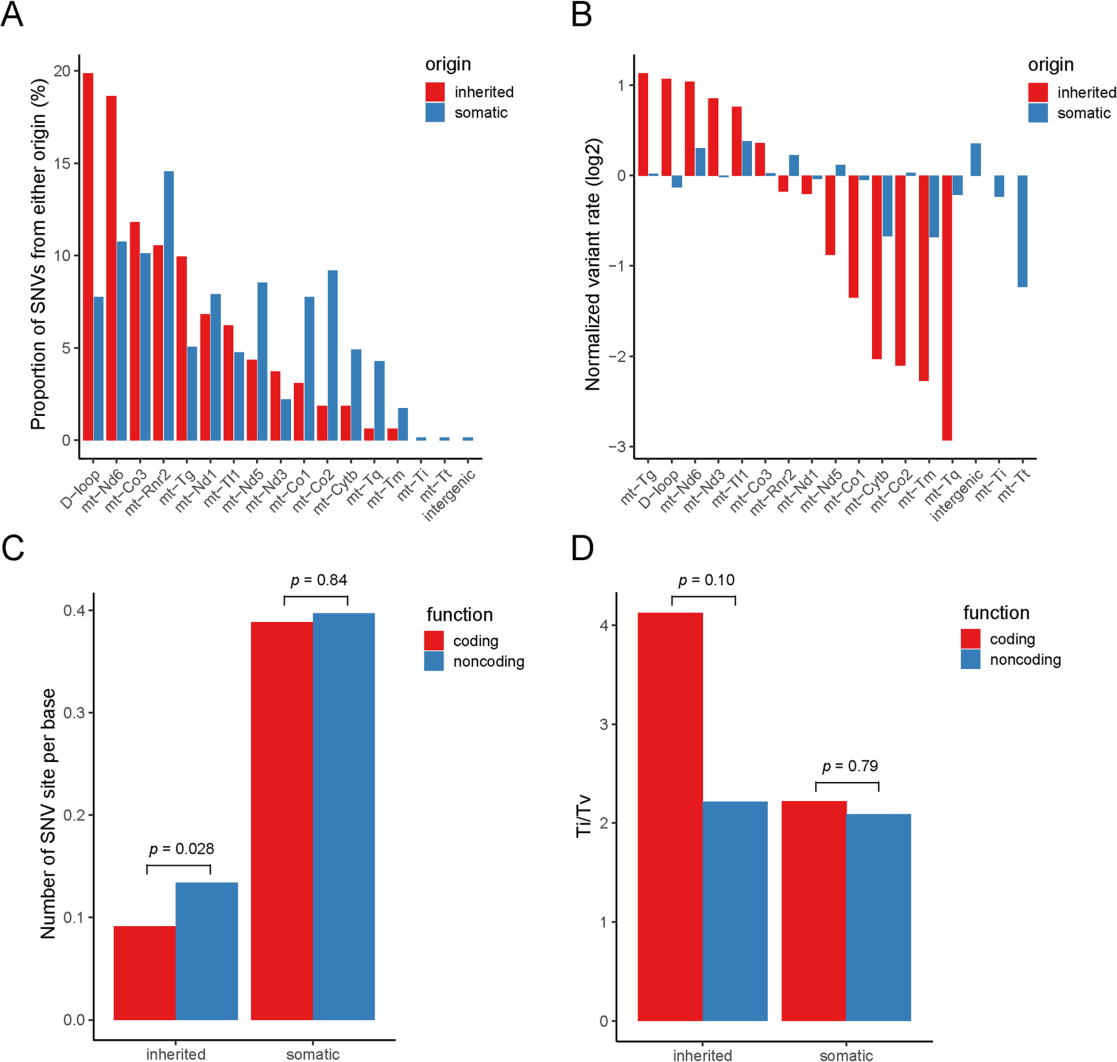
Mutational preference comparison between the inherited and somatic SNVs. **A** The proportion of inherited (red) and somatic (blue) SNVs within target region (gene loci). The percentages were calculated within each SNV class: 161 for inherited and 632 for somatic. **B** Length-normalized mean-centered variant rate for inherited (red) and somatic (blue) SNVs in each gene/locus. **C** The number of SNV sites per base in coding (red) and non-coding (blue) regions for inherited and somatic group of SNVs. **D** The Ti/Tv ratio of inherited and somatic SNVs classified by coding (red) and non-coding (blue) regions. Analyses were performed on data from *n* = 13 mouse pups, *n* = 102 cells, and *n* = 1645 single mitochondria

We next compared inherited and somatic SNVs for their mutational pattern. The two classes showed distinct regional preferences for transitions (Ti, purine to purine or pyrimidine to pyrimidine change) and transversions (Tv, purine to pyrimidine change or vice versa). Inherited SNVs occurred most frequently in D-loop (20%) and mt-Nd6 (19%), while somatic SNVs occurred most frequently in mt-Rnr2 (15%); only 8 and 11% somatic SNVs were found in D-loop and mt-Nd6, respectively (Fig. 3A). Somatic SNVs within mt-Co1, mt-Co2, mt-Tq, and mt-Tm were detected two times more often than inherited SNVs. To control for the PCR coverage for each region, we normalized the SNV occurrence per mitochondrion by the actual number of bases targeted by SMITO within each gene and defined it as the per-base variant rate. The somatic variant rate ranged from 0.16 to 0.51 while the inherited variant rate ranges from 0.02 to 0.23 (Additional file 10: Figure S7), derived by the absolute number of SNVs (~ 4 times more somatic than inherited SNVs). We observed a stark difference in the variant rate distribution (Fig. 3B). Inherited SNVs exhibited an uneven distribution when compared to the average, variants were more prevalent in mt-Tg, D-loop, mt-Nd6, mt-Nd3, and mt-Tl1 but less prevalent in mt-Tq, mt-Tm, mt-Co2, mt-Cytb, and mt-Nd5. In contrast, somatic SNVs showed a more uniform distribution as expected; only a modest decrease was seen in mt-Tt (Fig. 3B). The fact multiple animals have the same SNV, despite germline bottleneck, suggests some kind of maintenance mechanism.

We compared the per-base variant rate for inherited and somatic SNVs in both coding and non-coding regions (Fig. 3C). A marginal significance (*p* = 0.028) was observed between coding and non-coding regions for inherited group SNVs but not for somatic ones, confirming the observation in Fig. 3B that somatic SNVs had more uniform, functionally irrespective distribution. Similar comparison between coding and non-coding regions was made for Ti/Tv (Fig. 3D). Although neither showed *p* < 0.05, Fisher’s combined *p* = 0.015 hinted difference between the two classes of SNVs.

Ti/Tv ratios were compared for two classes of SNVs, revealing significant differences based on target regions and functional categories (Additional file 10: Figure S7C-D). Inherited SNVs displayed higher Ti/Tv ratios in mt-Co2, mt-Cytb, mt-Nd3, mt-Rnr2, mt-Tl1, and mt-Tm, indicating a prevalence of transitions. Conversely, somatic SNVs generally exhibited Ti/Tv ratios < 2.5 in these regions. However, mt-Tq, mt-Nd1, mt-Tg, mt-Nd5, and mt-Co3 showed inherited SNVs with Ti/Tv ratios twice as high as somatic SNVs. Notably, mt-Co1 was the sole protein coding gene displaying a fourfold higher Ti/Tv ratio in somatic compared to inherited SNVs. Fisher’s exact tests revealed that inherited SNVs had higher Ti/Tv ratios in rRNA and tRNA (*p* = 0.001 and 0.02, respectively), while somatic SNVs had higher Ti/Tv ratios in the D-loop (*p* = 0.002) (Additional file 10: Figure S7D). On comparing the Ti/Tv values from the polymorphisms from between -genera, -species, -strains (Mouse Genomes Project), and -population data (Additional file 11: Table S1, Additional file 12: Figure S8 A-B), we observed a clear trend for the whole mt-DNA (Additional file 12: Figure S8C): (1) phylogenetically more distant mt-genomes showed lower Ti/Tv, (2) D-loop had the lowest Ti/Tv while synonymous polymorphisms had the highest Ti/Tv. When limiting the polymorphisms to the SMITO assayed regions (Additional file 12: Figure S8D), we observed again low Ti/Tv values in nonsynonymous and D-loop polymorphisms but elevated Ti/Tv in synonymous polymorphisms in between-genera, -species, -population (see Additional file 13: Dataset S4). We used the McDonald-Kreitman test on mouse population data to test if evolutionarily selected genes show differential Ti/Tv from neutral genes. However, no gene showed significance for selection (Additional file 14: Dataset S5).

### Hierarchical distribution of the AF variation and heteroplasmy of inherited SNVs

**Fig. 4:**
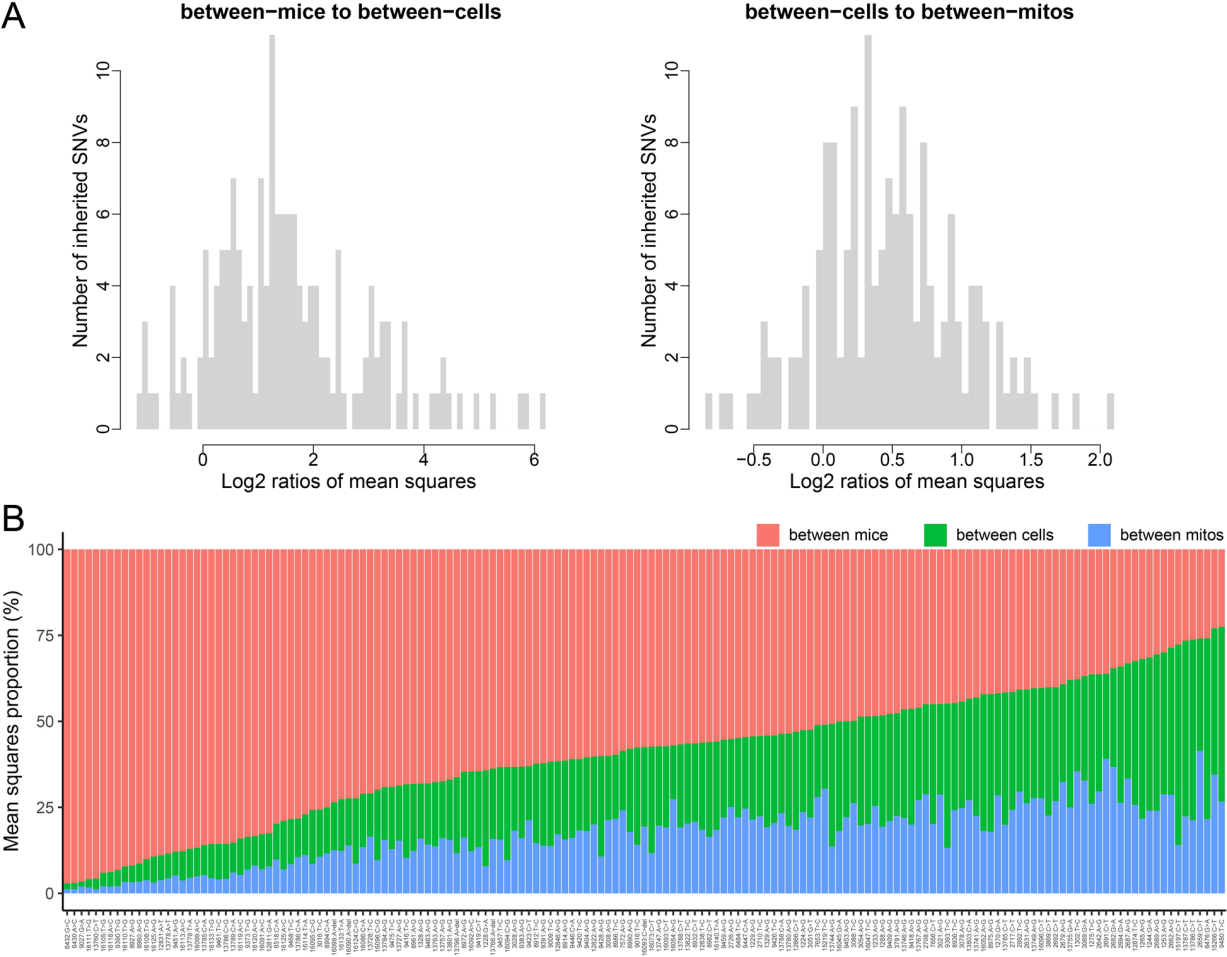
Comparison of the inherited SNVs’ allele frequency (AF) variation across mouse, cell, and mitochondria level. **A** The distribution of the ratios of: between-mice to between-cells variation (left) and between-cells to between-mitochondria variation (right). **B** The proportion of between-mice (red), between-cells (green), and between-mitochondria variation (blue) for each inherited SNV depicted on the *x*-axis. Analyses was performed on data from *n* = 12 mouse pups, *n* = 90 cells, and *n* = 1467 single mitochondria

New mutations in the mt-genome alter frequency through replication, drift, and selection. AF changes may occur due to unequal nucleoid replication within a single mitochondrion, uneven genome segregation between mitochondria (fission/fusion), segregation of mitochondria during cell division at the cell level, and germline transmission of inherited SNVs at the animal level. Thus, AF can be modulated at multiple levels. To investigate the variation of AF from a hierarchical perspective, we estimated the AF variation for inherited SNVs at three levels: between -mice, -cells, and -mitochondria using a nested ANOVA model after arcsine transformation (for details see “Methods”). To make variations across levels comparable, the mean-of-squares (MS) were measured. Most of the SNVs showed larger (average of 3 times) variations at the mouse level than the cell level (Fig. 4A, left), and a larger (average of 1.4 times) variation at the cell level than the mt level (Fig. 4A, right). These systematic biases are unlikely to be experimental or biological noise (Additional file 15: Figure S9) since random noise is expected to result in an equal MS across all three levels. The mouse and the cell identities were shuffled to generate a null distribution of the MS at the three levels. The null distribution level showed an average of 1/3, while the mouse level showed twice the amount of AF variation (Additional file 15: Figure S9A). SNVs according to the between-mice variation were plotted in a descending order (Fig. 4B). SNVs with the top between-mice AF variation include 6430:G > C, 6432:A > C, and 9027:G > A (Additional file 15: Figure S9B) whereas SNVs 2659: C > T, 15266: C > T and 1253: A > G SNVs exhibit larger inter- and intra-cellular AF variance. These data suggests that heteroplasmy observed for different sites may be a result of site-specific selection.

Often unsupervised dimension reduction is used to highlight sample similarities and differences in large cohorts of data and was recently used to assay mitochondrial variants in single-cell RNAseq from human patient samples in Wang et al. [31]. We however do not find compelling low-dimensional discrimination (Additional file 16: Figure S10) due to the following factors: (1) unlike the Wang et al. data, our individual mitochondrion samples are from inbred mouse single cells rather than a random population of human sample and due to less genetic diversity, our dataset has lower allele frequencies which results in lower discrimination with respect to standard genetic metrics, (2) our data is from the mouse, which has a higher mt-genome mutation rate as compared to humans [32], and (3) there is a higher allele frequency variation across mouse cells [22] which complicates the identification of distinct clusters. We further note that low-dimensional visualization of genetic data has considerable non-intuitive problems as previously noted [33, 34] and typically direct statistical treatment is more representative of the data than dimension reduction.

### Comparison between astrocytes and neurons in the SNV incidence and AF variance

**Fig. 5:**
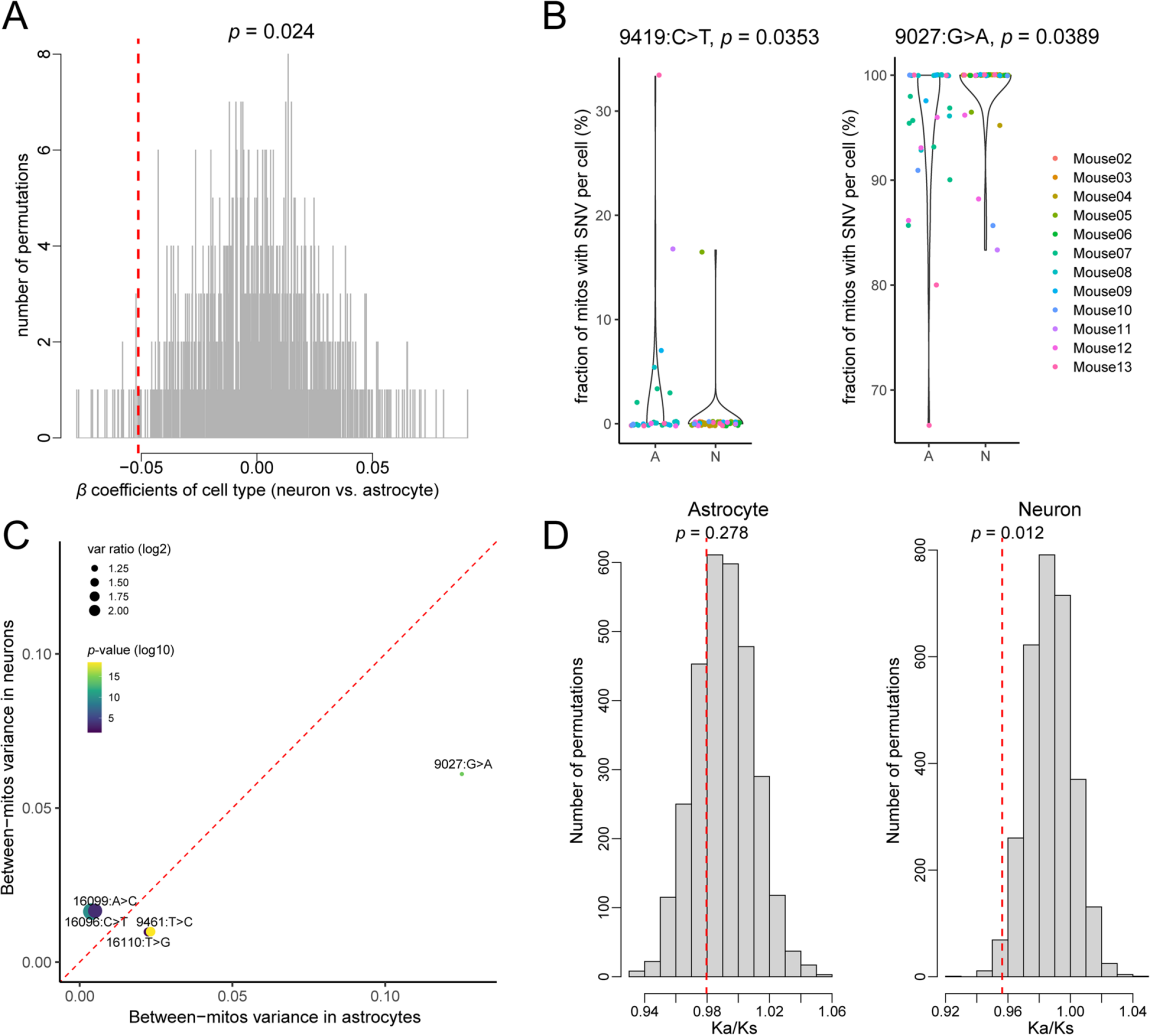
Cell type difference in the SNV incidence and allele frequency (AF) variance. **A** The null distribution (gray lines) and the observed value (red dashed line) of the cell-type effects on the number of total SNVs per mitochondrion. **B** The percentage of SNV-carrying mitochondria per cell for astrocytes “A” or neurons “N” for 9419:C > T (left) and 9027:G > A (right). **C** The scatterplot depicting the between-mitochondria variance in neurons on the *y*-axis to the between-mitochondria variance in astrocytes on the *x*-axis. The size of circles indicates the ratio of the variance (absolute value of log2), and the color gradient indicates the *p*-value (minus log10) from F-test (two-sided). **D** Ka/Ks statistics in astrocyte SNVs, and neuron SNVs. The red dashed line represents the observed Ka/Ks in respective cell type. Analyses was performed on data from *n* = 12 mouse pups, *n* = 90 cells, and *n* = 1467 single mitochondria

Unlike neurons, astrocytes are mitotic and are known to have more reactive oxygen species due to lower mitochondrial bioenergetic efficiency [25]. We hypothesized that astrocytes would exhibit more SNVs than neurons. On comparing the number of SNVs presented in the two cell types, we observed a mild yet significant difference (*p* = 0.024, for details see “Methods”) between the two cell types, with neurons showing ~ 5% lesser number of total SNVs when compared with astrocytes (Fig. 5A). We further investigated if there was cell-type difference in the SNV occurrence for specific SNV(s). By fitting the presence or absence of an SNV to a logistic regression model (the mouse effect included as the covariate), we found that 9419: C > T occurred more frequently in astrocytes and 9027: G > A more frequently to neurons with significances of *p* = 0.035 and 0.038, respectively (Additional file 17: Dataset S6). As an independent test, we compared the fraction of SNV-carrying mitochondria between the two cell types controlling for the mouse effect and we were able to recapitulate significance of these two SNV differences (*p* = 0.036 and 0.052, respectively) (Fig. 5B). To understand the functional consequences of 9419:C > T which impacts the first base of the Ac-loop of mt-Tg [35], we used RNAfold [36, 37]. RNAfold predicted this mutation to cause the minimal free energy to decrease from − 6.60 kcal/mol to − 8.60 kcal/mol, which may result from an additional A:U pair preceding the anticodon (Additional file 18: Figure S11). We found that 9027:G > A showed twice as much between-mt AF variance in astrocytes than in neurons (Fig. 5C). The cell-specific differences in the percentage of mitochondria with certain SNV site alleles (9419: C > T and 9027: G > A) emphasize differences in maintenance of SNVs for mt-genome between neurons and astrocytes.

To further investigate if evolutionary constraints on mt protein coding genes were different between neurons and astrocytes, we performed Ka/Ks analysis which takes into account the ratio of the number of nonsynonymous substitutions per nonsynonymous site (Ka) to the number of synonymous substitutions per synonymous site (Ks). Ka/Ks = 1 suggests neutrality, < 1 suggests purifying selection and > 1 suggests positive selection. We also found a reduced Ka/Ks relative to the background distribution (*p* = 0.04), indicating an overall purifying selection (Additional file 19: Figure S12 and Additional file 20: Dataset S7). Interestingly, astrocytes did not show significance for purifying selection whereas neurons did (*p* = 0.01). A recent study by Hu et al. [38] shows that neuronal cells are under strongest evolutionary constraint and higher selective pressure as compared to the other somatic cells including astrocytes, by single-cell RNA seq data analysis. Since neurons are the functional units of the nervous system and have limited regeneration, have a high energy demand, and are post-mitotic, the selection pressure experienced by these cells is higher than compared to mitotic cell types like astrocytes (Fig. 5D). But we also note that since neurons are post mitotic cells, the effective population size of non-dividing cells might be smaller than dividing cells and hence genetic drift in neurons may influence the changes in allele frequencies.

### Linkage of SNVs in the mt-genome

**Fig. 6:**
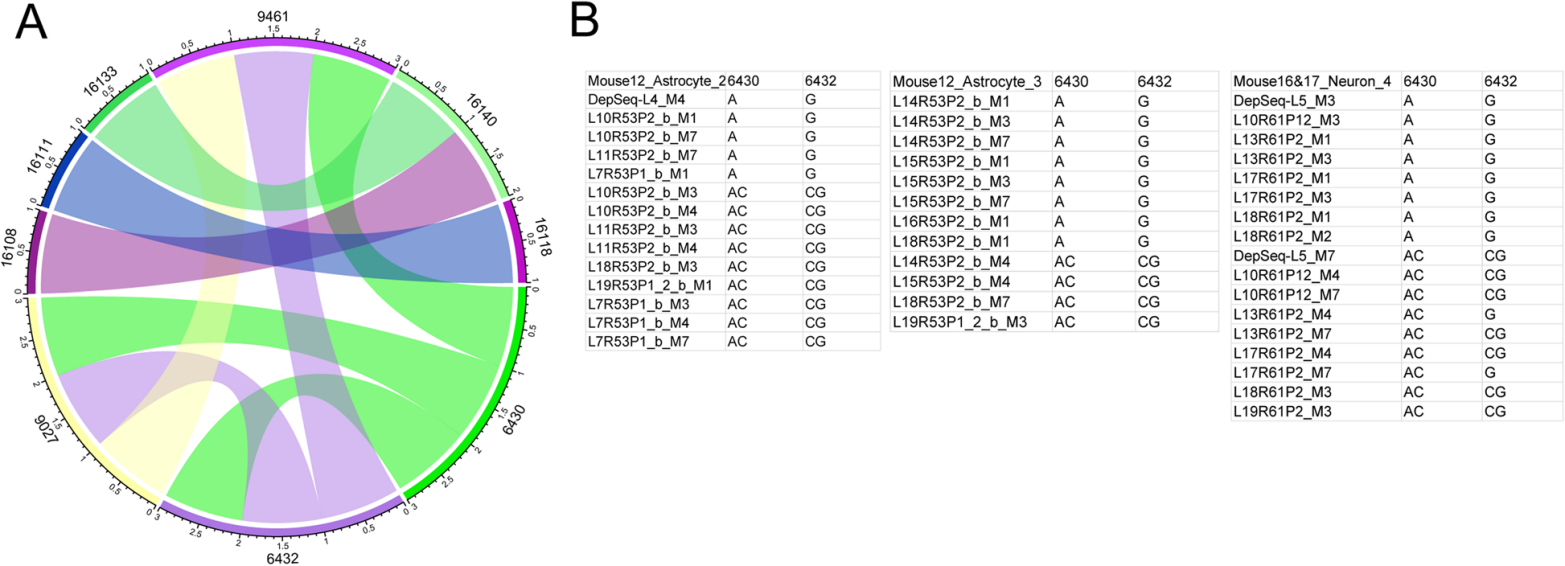
Linkage analysis of mt-SNVs. **A** Circle chord graph depicting significantly linked SNV loci, BH corrected *p-*value < 0.1. Analysis was performed on data from *n* = 13 mouse pups, *n* = 102 cells, and *n* = 1609 single mitochondria. **B** Single-cell level significant linkage 6430–6432 with genotype shown in each mitochondrion from each of 3 individual cells. Analysis was performed on data from *n* = 9 mouse pups, *n* = 26 cells, and *n* = 843 single mitochondria

We identified 9 pairs of SNVs within the target regions showing significant (BH adjusted *p* < 0.1) linkage across all cells and mice at position 6430, 6432, 9027, 9461, 16108, 16111, 16118, 16133, and 16140 (Fig. 6A), most of which were found within the D-loop region. Interestingly, two high AF SNV sites 9027 and 9461 showed strong linkage (Fisher’s exact test *p* = 8.82e − 5). Linkages can result from (1) co-segregated mitochondria or (2) an absence of recombination. Some short-range linkages such as 6430–6432 shared at single-cell level, in 3 cells from mouse 12 and 16–17, were caused by the lack of recombination, as the variant alleles were located at the same read (haplotype) (Fig. 6B, Additional file 21: Figure S13).

## Discussion

This study addresses the mt-SNV variance at the mouse, cellular and single-mt levels, heteroplasmy of specific SNV loci, cell type differences, and SNV segregation in the mouse neurons and astrocytes. There are differences in the prevalence or distribution of variants from specific regions of mt-genome because of their potential impact on the mitochondria or the cell. Given that we only targeted ~ 1/10th of the mt-genome, the number of SNV sites per mitochondrion found were relatively high compared to our previous study. This may be a result of larger sample size (~ 13 fold) at the level of individual mitochondria. This may also indicate that besides D-loop, there are certain regions in the mt-genome like rRNA/tRNA (as compared to the other target regions analyzed) that are more permissive to a nucleotide change. Previously, single-cell mtDNA mutation analysis in lymphocytes and monocytes from a 76 year-old female revealed that 70% of the mtDNA mutations were non-synonymous [39]. Similarly, we found that the 12 target regions analyzed had greater number of non-synonymous SNVs (~ 70% of these were transitions) as compared to the synonymous ones. Our comparison with mt sequences from related mouse strains and species suggests that a large fraction of our SNV sites (evolutionarily shared 89 sites) might have been ancestrally inherited, while the rest of SNVs might be de novo mutations whose effects might be recessive to other nucleoids in the same organelle, which explains the overall high proportion of the nonsynonymous changes in the target regions we analyzed.

Three SNVs were shared by all 13 mice: namely, 9027:G > A (average AF 86.3%), 9461:T > C (average AF 96.9%), and 12831:A > T (average AF 2.2%). Site 9461: T > C elicits a change in the start codon (AUU to AUC) for mt-Nd3 [36]. 9027:G > A causes a missense (glycine-to-serine) change in mt-Co3 and 12831:A > T results in premature truncation of mt-Nd5 (after ~ 60% of mRNA has been translated). These three sites show SNVs common to all sampled animals, despite germline bottlenecks in each generation, suggesting that they are actively maintained among mice. At 9461 position, two strains (PWK/PhJ and C57BL/6 J) use T while 15 others use C. We previously found that this site is also polymorphic among mouse strains [22], which was hypothesized to be cryptic heteroplasmy obscured by bulk sequencing. Thus, we continue to reason that 9461 is ancestrally polymorphic and under balancing selection in macro-evolutionary scale.

The overall nonsynonymous-to-synonymous ratio is approximately 2, suggesting a large fraction of the observed SNVs are randomly distributed over codon positions and transiting through purifying selection or are selectively neutral. Synonymous SNVs showed the highest transition-to-transversion (Ti/Tv) ratio (~ 10), which may occur as a result of wobble position degeneracy and/or RNA secondary structure constraints [40]; tRNA and rRNA showed high Ti/Tv (3.8 and 2.8, respectively), while D-loop showed the lowest Ti/Tv (~ 1). The difference of Ti/Tv between tRNA, rRNA, and D-loop possibly indicates different tolerance to changes in the thermal energy of base pairing due to the requirement of rRNA structure for translation while the D-loop region is gene regulatory and may be permissive to transversions that may promote regulatory protein binding to DNA. The observed transition bias (Ti/Tv of 2.72) in the mouse mtDNA is in agreement with previous studies [41–43]. Apart from the protein coding regions analyzed, the higher transition bias for the rRNA and tRNA target regions suggests that purification selection is not limited to protein coding regions in mouse mtDNA. However, since the transition transversion ratio varies between species, it will be interesting to see if Ti/Tv ratio for human mtDNA has significant differences between regulatory regions versus protein coding regions.

We found that the variants in the coding regions classified as “inherited” were mostly biased towards transitions over transversions. Interestingly, we found that that after the D-loop, which is known to be hypervariable, the number of transversions was highest in the mt-Nd6, a large fraction of which were non-synonymous. We found that SNVs in ND6 were predicted to have a higher impact when compared to other regions analyzed as noted in Table 2. Intriguingly, a study in 2013 by Bannwarth et al. reported ND6 and ND5 were the two protein coding genes in the mt-genome that had the most mutations in patients with mitochondrial diseases [44]. Being a crucial component for oxidative phosphorylation (OXPHOS), mt-Nd6 SNVs exhibiting a deleterious impact may result in a need for increased replication and transcription as a compensatory response, as observed in mt complex I from human skeletal muscle [45]. Thus, deleterious sequence variants within a gene may lead to clonal expansion of “imperfect” mt-Nd6 or complex I genes until a threshold is reached, as a result of compensatory increase in mtDNA replication [46, 47].

The hierarchical extent of overall variation shows the greatest variation between animals, followed by between cells, and the least variation manifest between mitochondria within a cell. This most likely reflects mainly neutral drift following the overall demographic structure, where the mt lineages have the highest separation between animals, then cells, then organelles within the same cell. At the level of cells, additional homogenizing forces can reduce variation such as suggested by Twig et al. that in mouse islet cells mitochondrial fusion and fission are coupled events [48]. Our data also suggests that there are some SNVs that show negligible variance at the mouse level but exhibit large variance between mitochondria within a cell, i.e., heteroplasmy. Like the codon position-specific selection pressure observed in mtDNA germline variants [49], this points to a region/gene loci-specific mechanism of SNV maintenance in a cell lineage for particular regions of mt-genome. The differences in mt-SNVs variance at cellular vs mt level at specific sites are important to consider for therapeutic strategies. In diseases with increased cellular variance of pathogenic SNVs, targeting and eliminating cells with higher mt-SNV prevalence could be a potential treatment.

The mitochondrial metabolic pathways in neurons and astrocytes are prioritized by their respective key function in the central nervous system, i.e., neurotransmission by neurons and support homeostasis in neurons by astrocytes. Several studies have shown that a main mt difference is that neurons are primarily dependent on OXPHOS to meet their high energy required for neurotransmission; however, astrocytes are primarily dependent on glycolysis. As a result of this main distinction, there are consequential differences in the bioenergetics, maintaining ROS, and signaling. An example of this is prioritizing conversion of pyruvate to lactate in order to shuttle lactate to neurons (since neurons are extremely efficient in utilizing lactate for OXPHOS) [25, 26, 50]. Since these two cell types are heavily interdependent, it is understandable that dysfunction in one cell type will impact the functioning of the other and has implication in numerous neurodegenerative disorders [51–53]. In this study, we have showed that there are cell type-specific differences in the ways mt-SNVs accumulate and propagate in these cells. The greater number of SNV sites in astrocytes (mitotic) than neurons (nonmitotic) is in agreement with the concept that mitotic cells tend to acquire more somatic mt mutations [46] and that astrocytes tend to have higher reactive oxygen species (ROS) production as compared to neurons [50, 54]. Given the subpopulation of mitochondria and specific target regions analyzed in the present study, it is likely that this difference will be more pronounced when the entire mt-genome is screened. Mt-SNV 9027: G > A is prevalent in multiple mouse strains, but no cell-specific differences have been previously reported. This study demonstrates for the first time that even in a wildtype mouse pup, there exist differences in the variance of allele frequencies and levels of heteroplasmy of certain SNV sites between neurons and astrocytes. For both sites 9419: C > T and 9027: G > A, the mitochondria analyzed in a neuron showed less variability as compared to astrocytes. This may be explained by segregation during mitosis in astrocytes which is absent for post-mitotic cells including neurons as well as stronger purifying selection noted in neurons (Fig. 5D). The purifying selection of mt-SNVs in neurons as shown by the Ka/Ks statistic suggests that neurons are under stronger evolutionary constraint as compared to astrocytes. Functionally, 9419: C > T is a mutation in tRNA glycine which leads to a RNA structural change as predicted by RNAfold (Additional file 18: Figure S11) and 9027: G > A is a missense mutation that changes the codon to serine from glycine of mt-Co3 [22]. It is interesting that both these SNVs are linked to glycine especially since glycine is an important part of biochemical pathways (glycine synthesis and metabolism, glutathione synthesis, glutamate-glycine cycle) in astrocytes [26] and also can act as an inhibitory neurotransmitter in neurons [55]. It is likely that there are differences in the tRNA pool availability for neurons vs astrocytes. The 9027: G > A in mt-Co3 (an important component of OXPHOS) observed more frequently in neurons may suggest some advantage to mitochondria since neurons are known to critically depend on OXPHOS for energy. Moreover, tRNA for serine requirement (due to glycine to serine change) is met more easily in neurons than in astrocytes. These findings suggest that the same mt-SNV sites may undergo different mechanisms of development, maintenance, and/or selection. Given that postnatal wildtype cells exhibit these differences, it suggests that the distinctive functions of mitochondrial bioenergetics may inherently be reflected at the mtDNA level and may potentially become pronounced during disease pathology or aging processes. Mean allele frequency differences for other SNVs were notable, but significance was lost after corrections for multiple tests.

Co-segregation in mt-DNA has been reported in human patient samples and trans-mitochondrial cybrids (created by fusion of enucleated cell with another mt-DNA depleted cell with a nucleus to study specific interaction between the nucleus and mt-genome) during aging, onset of deafness, and mt myopathy [56–58]. While a variant by itself may not be able to elicit a bioenergetic change, together with other mutations, these SNVs may contribute to inefficient mt functioning [57]. Of the most prominent linkages in the wildtype C57BL/6 mouse strain was that of 6430: A > C with 6432: G > C (both sites within mt-Co1) resulting from a lack of recombination. The linkage at 9027: G > A in mt-Co3 with 9461: T > C in mt-Nd3 may suggest selective advantages due to plausible cis effects, which when disrupted lead to sub-optimal oxidative phosphorylation. Although there may be a mutational bias towards C <  > T and G <  > A substitutions that could potentially increase linkage, except for the 9027–9461 linkage, all other linkages are not transitions. For 9027–9461, we also analyzed a mouse oocytes Nanopore sequencing dataset [59] and found 9027:G > A and 9461:T > C were indeed on the same haplotype. Further, these findings suggest numerous SNVs that may be linked, and if so, this may be a consequence of selective advantages conferred at either the cis or trans level. Regardless of the mechanism, this implies that the influence of pathogenic or non-synonymous SNVs may be impacted by targeting their corresponding linked SNVs. Long read length sequencing on single-mtDNA will help assess the co-segregation status of sequences highlighted in the present study.

Previous studies analyzing mt-genomes from single cells as well as single mt-genomes from single cells have been limited by technical challenge in single mitochondrion isolation. Several findings pertaining to mt heteroplasmy have been at the organism [60], tissue [20], or at the cell level [59, 61, 62]. However, hierarchical characterizations of mt variants at the single-mt level [22] are limited. In this study, we conducted an analysis of SNV from up to 50 individual mitochondria obtained from a single cell. Isolating homogeneous populations of mitochondria with specific characteristics is challenging due to this inherent heterogeneity. As we have examined multiple mitochondria but only a small fraction of the total mitochondrial population in a cell, it is probable that our analysis has targeted a subset of the population. This analysis was not limited by the isolation yield, but rather by the manual processing of single-mitochondrion samples (mtDNA amplification) on the day of isolation. However, in the future to increase the total throughput through single mitochondrion isolation and amplification method reported here, automatic liquid and a single-cell single-mitochondrion barcoding scheme will be developed and implemented.

## Conclusions

Despite limitations arising from the small number of analyzed cells per animal, these data provide valuable insights into variation in the single-cell mitochondrial DNA SNV landscape. The single cell–single mitochondrion approach (1645 individual mitochondria) in our study reveals higher number of SNV sites per mitochondrion than previously reported attributable to increased experimental resolution. The single-mt SNV landscape suggests that different loci within the mt-genome are subject to different mechanisms of clonal expansion and segregation. The purification selection in mouse neurons and with some of the cell type differences being modest, it will be interesting to determine whether these differences are present and expanded during human neurological disease development. These data are fundamental to the understanding of how different variants can exhibit site-specific differential patterns of clonal expansion and inter- and intra-cellular variation, which narrows the knowledge gap in understanding the functional role of heteroplasmy in disease processes.

## Methods

### Cell culture

Based on the protocol outlined by Kaech and Banker [63], some modifications were made for culturing primary cortical neuronal co-cultures. Briefly, the postnatal day 1 mouse pups (C57BL/6) were dissected to carefully isolate the cortex in cold Hank’s Balanced salt solution without Ca^2+^, Mg^2+^. The cells were then dissociated with 0.5% trypsin/EDTA in PBS followed by pipetting, trituration and passing the cell suspension through a cell strainer. Subsequently, the cells were plated on poly-D-lysine/laminin coated 12-mm coverslips in a 35-mm plate after live cell counting with trypan blue to achieve plating density of 200,000 cells/ml. Co-cultures of neurons and astrocytes were maintained in MEM with B27 supplement and 1% FBS at 37 °C in a 5% CO_2_ incubator as previously mentioned in Morris et al. [22].

### Staining and isolation of single cells

Dispersed mouse cortical neurons and astrocytes were seeded on the coverslips in a 35-mm dish which were stained with 100 nM of MitoTracker Deep Red FM (Invitrogen, M22426) at 37 °C for 20 min in an incubator (5% CO_2_) at DIV (days in vitro) 3, 7, or 21 days. The cell media with MitoTracker Deep Red was replaced with fresh media and incubated for another 20 min. A single neuron or astrocyte was harvested using a micropipette from a coverslip based on morphology (see Additional file 22: Figure S14 for representative images) with MitoTracker labelled cells under the microscope (× 40 magnification, Olympus IX70), and then was collected in the 1.7-mL Eppendorf tube with 4 μl of cell lysis buffer. After spinning down briefly, the Eppendorf tube was incubated on ice for 10 min followed by addition of 15 μl mt buffer (220 mM sucrose, 68 mM mannitol, 10 mM KCl, 5 mM KH_2_PO4, 2 mM MgCl_2_, 0.5 mM EGTA, PH 7.2; 0.01% bovine serum albumin and 1X Protease inhibitor cocktail) to the Eppendorf tube. The single neuron or astrocyte lysates were then used for preparation of the flow cytometry sorting samples.

### Morphology criteria for single-cell pickup

Since both neurons and astrocytes can show variable morphologies, we used the following criterion for each cell type. Neurons—pyramidal (A), bipolar (B) or unipolar (C) neurons, were selected (Additional file 22: Figure S14 A-C). A cell was classified as a neuron if it had a small soma (approximately < 10 µm) with elongated 1–3 processes. Astrocytes—Cells displaying “classic-star-shaped” morphology with multiple processes were chosen as astrocytes. A cell with a relatively larger (> 10 um) and flatter cell body, accompanied by more than 4 processes of similar lengths were designated as astrocytes (Additional file 22: Figure S14 D-F). Additionally, we have incorporated references demonstrating that the neurons and astrocytes chosen for analysis exhibited morphological characteristics consistent with those of mouse cortical neurons and astrocytes identified by immunofluorescent markers. Please refer to the following references showing neuronal and astrocyte morphology that resembles the cells we have selected as mouse cortical neurons and astrocytes respectively: Hilgenberg and Smith [64], Sciarretta and Minichiello [65], Granger et al. [66], Xu et al. [67], Baldwin et al. [68], Sathe et al. [69], Sosunov et al. [70], Sun and Jakobs [71], Purvis et al. [72], Sidoryk-Wegrzynowicz et al. [73], Sun et al. [74], to name a few.

### Conjugation of anti-TOMM20 antibody on microbeads

The conjugation of anti-TOMM20 antibody (Recombinant Anti-TOMM20 antibody [EPR15581-39]—BSA and Azide free, Abcam Cat# ab220822, RRID:AB_3097753) to microbeads through an avidin–biotin bond was modified based on the commercial protocol (Spherotech Inc., Particle coating procedures—https://www.spherotech.com/technical%20notes/STN-1%20rev%20C.pdf). The anti-TOMM20 antibody was biotinylated via Miltenyi One-Step Antibody Biotinylation Kit (Miltenyi Biotec, 130–093-385) following the manufacturer’s protocol. Ten micrograms of anti-TOMM20 antibody was diluted to 100 μg/mL by adding 1X PBS, followed by addition of the 10 μg of antibody to the well containing Miltenyi lyophilized biotinylation mix. After pipetting thoroughly, the mixture was incubated at room temperature for 24 h and stored at 4 °C before use. For coating microbeads, 2 μg of biotinylated anti-TOMM20 antibody was suspended in sodium phosphate buffer (PB buffer, 0.1 M, pH = 5.5) at 10 μg/mL. Twenty million avidin-coated polystyrene particles (1.7–2.2 μm, 0.1% w/v, Spherotech Inc, VFP-2052–5) were added and incubated with biotinylated antibody for 1 h at room temperature on rotator covered by aluminum foil. After incubation, the coated microbeads were centrifuged at 150,000* g* for 10 min and resuspended in 0.4 mL PB buffer. The centrifugation of beads was repeated, and the beads were resuspended in 0.4 mL of filtered mt buffer mentioned in the previous section. Anti-TOMM20 antibody coated beads were freshly prepared each time for mitochondria isolation.

### Flow cytometry of captured mitochondria from single cells

Anti-TOMM20 antibody conjugated fluorescent microbeads (1–2 million—10 times of the estimated number of mitochondria from individual cells) were added to the single neuron or astrocyte cell lysate and the final volume of each sample was adjusted to 100 μL by adding filtered mt buffer. After an hour of incubation at room temperature on a shaker, the product was transferred to flow tubes for FACS sorting. Flow cytometry was performed on a LSR II flow cytometer (BD Biosciences) and BD FACS Aria II (BD Biosciences) respectively for assay validation and sorting at the Penn Cytomics and Cell Sorting Shared Resource Laboratory (RRID:SCR_022376). Flow cytometric analysis was performed using FlowJo software (Additional file 23: Figure S15).

### Amplification of mt-genomes by Rolling Circle Amplification (RCA)

Single mitochondria were sorted in each of the wells of a 96-well plate containing 4 μl of mild lysis buffer (1 × TE, 0.1 M NaCl, 2 mM CaCl_2_). One microliter of 0.25 μg/μl Proteinase K (Roche, 03115879001) was added to each well and incubated at 55 °C for 30 min. The Proteinase K inactivation was performed by incubation at 95 °C for 10 min. The plates were rested on ice until the next step. Nuclease-free water and 1X Phi 29 reaction buffer (New England Biolabs, B0269SVIAL) were added to each well followed by incubation at 70 °C for 3 min. The plates were immediately rested on ice for at least 2–4 min to complete denaturation. The samples were incubated at 25 °C for 50 min with random primers (20 μM). Two-millimolar dNTPs (Invitrogen, 18,427,088), 10 mM DTT (Thermo Scientifc,707265ML), and 5U Phi 29 DNA polymerase (New England Biolabs, M0269L) were added to each well to set up the reaction. The 25 μl reaction mixture was incubated at 30 °C for 32 h. Another 5U of Phi 29 DNA polymerase was added to the reaction mixture after 20 h to continue the reaction. The reaction was stopped after 32 h by incubation at 65 °C for 10 min. The RCA product was stored at − 20 °C until the next step. The amplified mt-genome was validated by ethidium bromide agarose gel electrophoresis (Additional file 24: Figure S16).

### Library preparation

For each library, 10 Non-Seq Barcodes (namely, M1 through M10, Additional file 25: Table S2) were designed for multiplexing 10 mitochondria. For each mitochondrion, 12 target regions were further PCR amplified using forward primers beginning with the aforementioned Non-Seq Barcodes and reverse primers as listed in Additional file 26: Table S3. These regions were selected based on the SNV sites reported in our previous paper [22]. All primers were designed through the NIH Primer-Blast tool (https://www.ncbi.nlm.nih.gov/tools/primer-blast) and synthesized by Integrated DNA Technologies. PCR amplification was performed with 1 μl of RCA product from each single mitochondrion sample, 1 μl of 24 oligos pool for 12 target region amplification (1.25 μM), and 23 μl Platinum PCR SuperMix (Invitrogen, 12532–016), using the following program: 94 °C 2 min for the 1st cycle, followed by 25 cycles of 94 °C 30 s, 49 °C 30 s, 68 °C 30 s, followed by addition of 15 μl Platinum PCR SuperMix for next 20 cycles using the same PCR program as the previous 25 cycles, with a final hold at 4 °C.

After the amplification, each PCR product was analyzed on the Agilent Type Station 4150 using a High sensitivity D5000 Screen Tape Assay. Then 10 barcoded single mitochondria were pooled together and cleaned up by 1.5X AMpure XP beads (Beckman Coulter, A63881). Fifty nanograms of the pool was input into a library using TruSeq Nano DNA kit (Illumina, 20015964) as per the manufacturer’s instructions. Twenty-three libraries were combined into a sequencing run and then sequenced by 1 × 150 bp single-end kit on Illumina NextSeq 500.

### Mitochondrial (mt) barcode demultiplexing

To determine the optimal threshold of error tolerance in mt barcode demultiplexing, the Levenshtein distance was calculated between any two barcodes (Additional file 27: Table S4), and a minimal distance 3 was obtained. Therefore, at most two errors in searching each of the 11-bp barcodes were permitted using the command: cutadapt -g XCGATTATCACG -e 0.2 -O 11 (CGATTATCACG being M1 as an example, Additional file 25: Table S2). For more details on sample size for the number of mitochondria isolated per cell from each mouse pup, see Additional file 1: Figure S1A and for PCR read depth for each mt barcode, see Additional file 28: Figure S17 and additional file 29: Figure S18.

### Out-of-range SNV filtering

The mt barcode and the PCR primers’ efficiency by the read depth was examined at each base position. We noticed the trailing coverage at the 3′ end of some PCR target regions (e.g., Regions 3, 6, and 7, Additional file 29: Figure S18). For the sake of rigor, the bases out of the sequencing read length were designated as “out-of-range,” SNVs (if any) inside these regions were discarded. In addition, SNVs (if any) inside the PCR forward primers were removed because the genetic variants could be masked by the identically synthesized primer sequences, and hence any SNVs inside the primers could be artifactual.

### Variant allele frequency threshold

Assuming that the RCA reaction went through ${n}_{1}$ cycles at a per-base error rate ${\varepsilon }_{1}$ and that the PCR reaction went through ${n}_{2}$ cycles at a per-base error rate ${\varepsilon }_{2}$. For a particular site, let ${X}_{1}$ be the total number of mutations resulting from RCA and that ${X}_{1}\sim \text{binom}\left({n}_{1},{\varepsilon }_{1}\right)$, hence after RCA ${X}_{1}$ mutant copies and ${n}_{1}-{X}_{1}$ intact copies will be obtained. Assuming no backward mutation, all mutated molecules would be amplified by PCR, and the final number of mutant copies would be ${{X}_{1}\times 2}^{{n}_{2}}$. For each of the RCA intact copies, assuming it induces ${X}_{2}^{i}$ errors during PCR cycle $i$, where there are ${2}^{i-1}$ reactions, hence ${X}_{2}^{i}\sim \text{binom}({2}^{i-1},{\varepsilon }_{2})$*,* and these errors would propagate and eventually result in ${2}^{{n}_{2}-i}\times {X}_{2}^{i}$ mutant copies. Therefore, the total number of mutant copies exclusive to PCR would be $({n}_{1}-{X}_{1})\sum_{i=1}^{{n}_{2}}{2}^{{n}_{2}-i}{\times X}_{2}^{i}$. After RCA and PCR, the total number of amplicons would be $N={n}_{1}\times {2}^{{n}_{2}}$ and among them, the total number of mutants would be $X={{X}_{1}\times 2}^{{n}_{2}}+({n}_{1}-{X}_{1})\sum_{i=1}^{{n}_{2}}{2}^{{n}_{2}-i}{\times X}_{2}^{i}$. Assuming the amplicons to the depth of $M$ were sequenced and $Y$ variant bases were observed, then $Y\sim \text{binom}\left(M, \frac{X}{N}\right)$, hence at the VAF threshold $\theta$, the probability of making a false positive call is $\text{Pr}\left(Y>M\theta \right).$

For simplifying $$\frac{X}{N}$$.

$$\frac{X}{N}=\frac{{{X}_{1}\times 2}^{{n}_{2}}+\left({n}_{1}-{X}_{1}\right)\sum_{i=1}^{{n}_{2}}{2}^{{n}_{2}-i}{\times X}_{2}^{i}}{{n}_{1}\times {2}^{{n}_{2}}}=\frac{{X}_{1}}{{n}_{1}}+\left(1-\frac{{X}_{1}}{{n}_{1}}\right)\sum\limits_{i=1}^{{n}_{2}}{2}^{-i}{\times X}_{2}^{i}$$

Assuming ${X}_{1}$ and ${X}_{2}$ are independent random variables, we further have

$$\text{E}\left(\frac XN\right)=\text{E}{\left(\frac{X_1}{n_1}\right)+\text{E}\left(1-\frac{X_1}{n_1}\right)\text{E}\left(\sum\limits_{i=1}^{n_2}2^{-i}{\times X}_2^i\right)=\varepsilon_1}+\left(1-\varepsilon_1\right)\sum\limits_{i=1}^{n_2}2^{-i}\times2^{i-1}\times\varepsilon_2=\varepsilon_1+{\frac12n}_2\left(1-\varepsilon_1\right)\varepsilon_2\approx\varepsilon_1+{\frac12n}_2\varepsilon_2$$

Given that ${\varepsilon }_{1}=5\times {10}^{-6},$ ${\varepsilon }_{2}=3.67\times {10}^{-6}$, ${n}_{2}=45$, we have $\text{E}\left(\frac{X}{N}\right)\approx 8.758\times {10}^{-5}$. Since the post-filtering minimal sequencing depth $M=50$ is sufficiently large and $\text{E}\left(\frac{X}{N}\right)$ is sufficiently small, we can approximate $Y$ with a Poisson distribution with $\lambda =4.379\times {10}^{-3}$. Therefore, at the VAF cutoff $\theta =1\%, 2\%, 5\%, 10\%$, the false positive rate to be $\text{Pr}\left(Y>M\theta \right)=4.370\times {10}^{-3}, 9.560\times {10}^{-6}, 1.395\times {10}^{-8}, 9.756\times {10}^{-18}$, was expected respectively. Given 1424 sites in total, the expected false positive sites were $6.222, 1.36 \times {10}^{-2}, 0,\text{ and }0$ at the corresponding cutoff. Hence 5% VAF threshold was chosen.

### SNV calling within each mitochondrion sample and Quality Control Procedure

We processed the raw reads using an in-house Next-Generation Sequencing (NGS) pipeline. First, barcoded reads from the same library were demultiplexed into individual mt reads. Then, sequencing adapters and poly-Gs were searched, trimmed, and reads shorter than 20 bp were discarded. Remaining reads were mapped to the mouse mtDNA (genome build mm10) using STAR,v2.7.2d (RRID:SCR_004463) [75] with default parameters except “–outFilterMismatchNoverLmax 0.1,” which made mapping more stringent than by default. To disable spliced alignment, additional arguments “–alignIntronMax 1 –alignSJDBoverhangMin 999” were used. Afterwards, only uniquely mapped reads inside the PCR regions were extracted, and for each PCR region, per-base read pileup was generated by samtools, v1.13 (RRID:SCR_002105) with parameters “–excl-flags UNMAP,SECONDARY,QCFAIL –count-orphans –min-BQ 0 –min-MQ 0 –reverse-del.” In case of memory exhaustion, an additional argument “–max-depth 500000” was used such that the maximum depth was limited to 500,000.

To prevent false positives caused by sequencing errors, polymorphic bases with Phred scores below 30 were discarded. This threshold was derived from the Phred calibration analysis, where all non-reference bases were collected and their theoretical (*x*-axis) error rate was plotted from Phred calls against the observed error rate (*y*-axis), which was composed of true SNVs and technical errors. As expected, two well separated clusters were observed: the top-right quadrant (VAF >  = 5%, Phred >  = 30) and the bottom-left quadrant (VAF < 5%, Phred < 30) (Additional file 30: Figure S19). The threshold was maintained at 30 for the sake of simplicity (not lowered to 28.99—the red dashed line). Only < 10% read depth was lost when imposing the more stringent cutoff, and most of the loss occurred to the 3′ end.

For accurate AF estimation, SNVs were called from positions with (1) read depth ≥ 50, and (2) VAF ≥ 5% (Additional file 31: Dataset S8). The PCR primer regions were discarded from the SNV calls. To reduce false positives, we discarded PCR primer regions from SNV calls or SNVs discordant between the alignment tool STAR [75] and BWA (RRID:SCR_010910) [76] (Additional file 32: Figure S20). Besides the original SNV call, another much more stringent SNV list was derived by the following filtering criteria: (1) reads must be on the positive strand only, (2) reads must start off the 5′ end of the PCR forward primer only by + / − 1 bp, and (3) read length must be ≥ 135 bp. The stricter SNV call verified 92.5% of the total SNVs from the original call.

### Control experiment assessment

The raw read depth in the single mtDNA samples versus different types of controls was compared: pooled mtDNA( +), pooled mtDNA( +) primer( −), pooled mtDNA( −) primer( +), pooled mtDNA( −)primer( −) in 12 PCR target regions (Additional file 33: Figure S21). As expected, in most of the amplified SNV regions, the positive controls (i.e., pooled mtDNA from mouse brain—double positive control) gave rise to the highest read depth, and the single-mitochondrion, double positive samples often showed the second highest depth. Compared with the positive controls, the negative controls generally showed two orders of magnitude lower depth. It is noteworthy that the read depth is not an accurate estimator for sensitivity for it is heavily influenced by PCR efficiency, starting material and sequencing depth, hence single-mitochondria samples and pooled controls might not be well comparable. The main purpose of this analysis was to highlight the global difference between the positive and negative pooled controls, and the results did match our expectation. The QC statistics from pipeline preprocessing can be found in Additional file 34: Dataset S9.

### Correction for the soft-clipped 9027

During read alignment, we noticed that STAR could soft-clip the mismatching base(s) at the 3′ end of reads and hence could cause an underestimated VAF should the variant base be at the 3′ end. A systematic diagnostic analysis was performed to evaluate the impact of this issue. We found that 9027 was the only position affected by this problem—of 1489 mitochondria that have base coverage at 9027 from raw reads, there were in total 107,259,677 As, 13,953,942 Gs, 330,682 Cs, and 294,080 Ts; STAR discarded the non-reference As. Therefore, the 9027:G > A read frequency for each sample was manually compensated. Additional file 35: Figure S22 represents the change in 9027:G > A VAF before and after the correction.

### NUMTs computational analysis on SMITO data

Given our single-cell fluorescence activated mt isolation procedure [23], we do not expect nuclear DNA contamination in our samples. In support of this argument that nuclear-embedded mitochondrial DNA sequences (NUMTs) [77–80] are highly unlikely in our dataset, we performed computational prediction of NUMTs. To determine the number of false positives due to the potential NUMTs [77–81] contamination, we first assembled a list of mouse NUMTs regions. Briefly, we synthesized 50-bp reads at each mitochondrial position and mapped them to the nuclear genome using BLAST with highly sensitive parameters: -num_alignments 1000 -word_size 6 -perc_identity 80 -gapopen 3 -gapextend 1 -evalue 1; the results were merged as disjoint regions and referred to as NUMTs. We synthesized 50 bp reads from NUMTs and mapped them back to the mt-genome using bwa mem. To make the mapping quality comparable to STAR, we filtered for alignments with MAPQ ≥ 60. Finally, we called 381 NUMTs original, false mt-SNVs (Additional file 36: Figure S23A and Additional file 37: Dataset S10) using the aforementioned procedure in the “Methods” section. Please note that, for this analysis, we only required VAF > 0 and did not filter out low-depth or low-VAF SNVs, hence our false positives here are overestimated. We compared our SNVs against this list; only 27 overlapped suggesting that, by computational analysis at most, 27 out of 838 (3.2%) SNVs could potentially be artifacts originating from NUMTs.

Furthermore, mapping reads that did not align to the mouse mt-genome to the mouse nuclear genome yielded a minimal number of unique alignments (average 0.33%, median 0.08%). Given the same amplification, library prep and sequencing conditions, nucleus borne reads should have mismatch rates comparable to mt reads. These nucleus exclusive alignments showed significantly (Wilcoxon rank-sum test *p*-value < 2.2e − 16) higher mismatch rate (mean 5.19%, median 5.27%) than mt exclusive alignments (mean 1.41%, median 1.17%).

To check if our single mitochondrion samples are contaminated with nuclear material, PCR was performed on the single mitochondrion RCA products using primers targeting a region from mouse mt-genome (Target region 6) and two independent regions in mouse chromosome 1 (Pet-1, FEV transcription factor) and chromosome 6 (Gapdh, Glyceraldehyde-3-phosphate dehydrogenase). Both 1 µl and diluted single mitochondria RCA product (2 µl of 1:25 dilution) was used to test the presence of mtDNA as well as nuclear contamination following the procedure mentioned in the main methods section under library prep. The annealing temperatures of 50 °C, 55 °C, and 57 °C were used respectively for target region 6, Pet-1, and Gapdh. The isolated mtDNA (0.1 pg) from mouse pup brain that had also undergone RCA was used as a positive control template (0.5 µl) for target region 6, and 12 ng of mouse genomic DNA from mouse pup brain was used as template for positive controls, Pet-1 and Gapdh. One microliters from final PCR mix of test samples and 0.5 µl of positive controls was loaded on Agilent D5000 ScreenTape. Additional file 36: Figure S23B is a representative image showing PCR product from 4 single mt samples (from two independent experiments #45 and #22 – well IDs are labelled on the gel picture H1, H2 from #45 and B3, B4 from #22) diluted 1_25 samples targeted for target region 6 (Lanes 2–5), Pet-1(Lanes 6–9), Gapdh (Lanes 10–13), positive control for target region 6(+ Mt—Lane 14), positive control for Pet-1(+ Nu—Lane 15), and positive control for Gapdh (+ Nu—Lane 16). The expected band for target region 6 is 189 bp, Pet-1 is 237 bp and Gapdh is 452 bp for mRNA and 646 bp for DNA. The primer information for target region 6 is mentioned in Additional file 26: Table S3. The primer information 5′–3′ for Pet-1 and Gapdh are as follows:

- Pet-1 Forward Primer – AGATTCTGGAACTCCCGTGT
- Pet-1 Reverse Primer – GAGAAAGGGAAGCCAGAGTG
- Gapdh Forward Primer – ACCACAGTCCATGCCATCAC
- Gapdh Reverse Primer – TCCACCACCCTGTTGCTGTA

### SNV impact annotation

The package Variant Effect Predictor, VEP—v102.0 (RRID:SCR_007931) [82] with the parameters “–offline –cache –species mus_musculus –force_overwrite –everything –i” was used to infer the consequence of the 1032 non-reference alleles based on the mouse mt codons. We manually labelled AUU-to-AUC (e.g., at 9461) as “synonymous” according to the mouse mt codon table. Besides, 3772:C > T hit both mt-Ti (on the plus strand) and mt-Tq (on the minus strand) while VEP predicted identical severity; only former was retained for simplicity.

### Mouse strains coordinate standardization

During the phylogenetic analysis, a subtle discrepancy was seen in the UCSC GRCm38 reference genome (C57BL/6 J) which is used in this study and the C57BL/6NJ genome (CM004277.1) from the Mouse Genomes Project: the former has 16,299 bp while the latter has 16,300 bp (Additional file 38: Figure S24). Hence, a distinction was made between C57BL/6 J and C57BL/6NJ: UCSC C57BL/6 J was added to the 16 strains from the Mouse Genomes Project and multiple sequence alignment was performed to 17 strains in total. The mitochondrial coordinate system was standardized based on UCSC GRCm38 (C57BL/6 J).

### Comparing SMITO and sites segregating among mouse strains

To assess the data quality, 17 mouse strains’ mt-genomes data from the Sanger Mouse Project [83, 84] were aligned (multiple sequence alignment) using Clustal Omega (RRID:SCR_001591) [85], which identified 1494 segregating sites. Among them, 128 sites within the 12 mt target regions analyzed in our study were termed as “17 mouse strains data” and compared to SMITO dataset. The overlap was quantified by hypergeometric tests, and by major allele or top minor allele consistency.

### Inherited-somatic SNV demarcation

To compute the null distribution of number of SNVs shared by *k* mice with animal specificity controlled, the SNVs in each mouse were shuffled such that the total number of SNVs remained identical in each permutation (*n* = 100), and the average was calculated as the expected number of SNVs shared by *k* mice. The expected and the observed curve were contrasted; the first cross (*k* < 1.9) was taken as the cutoff for somatic SNVs; inherited SNVs were taken only if *k* ≥ 3 for stringency.

### Mutational pattern in annotation groups

To compare mutation pattern for somatic and inherited SNVs, the total SNVs were divided into two groups: coding and non-coding. In each group, the per-base mutational rate and the transition-to-transversion (Ti/Tv) ratio was compared using Fisher’s exact test (two-sided).

### Hierarchical variation distribution of the inherited SNVs

ANOVA was performed on the VAF of 161 inherited SNVs, and the Mean of Squares (MS) was computed at three levels: the mouse, cell, and mitochondria. For each SNV, the ratio of the mouse-level MS to cell-level MS, and of the cell-level MS to mitochondria level MS was calculated. To ensure that the distribution of the MS was not skewed by noise (VAF < 5%), mouse and cell labels were randomized by 1000 permutations, creating a null distribution of the mouse-, cell-, and mitochondria-level MS for the observed distribution to be contrasted. For each SNV, the MS at three levels were finally normalized to a percentage. This analysis involves cell-level and mouse-level variance partition where mouse identity has to be ascertained, so the mixed pup culture (16&17) was excluded.

### Cell type comparison for SNV incidence and VAF variance

The number of SNVs per mitochondrion (log(*x* + 1) transformed) were compared in neurons and astrocytes by a mixed-effects linear model using the cell type as the main effect, the mouse ID as the random effect, and the number of bases with sufficient depth (log(*x* + 1) transformed) as covariates; the null distribution was built by 1000 permutations to the cell type label. Each SNV incidence between the cell types were compared using two independent methods: (1) logistic regression was performed to predict the presence or absence of an SNV in individual mitochondria using the cell type as the main effect and the mouse ID as the random effect; (2) the fraction of mitochondria with variant in each cell (excluding cells with < 3 mitochondria) was calculated and then fitted to a weighted logistic regression using the cell type as the main effect and the mouse ID as the random effect. R package lme4,v1.1–28 lme4 (RRID:SCR_015654) [86] was employed for the mixed-effects models. Finally, the between-mitochondria variance between the cell types for each SNV was compared. To control for the mouse and the cell effect, the VAF was arcsine transformed and ANOVA was performed using the mouse and cell ID as the covariates, and the MS of the model residuals from the cell level was taken as the between-mitochondria variance estimate. The analysis was performed for both cell types separately. Mouse16&17 is a co-cultured sample from two mice (mixed pup culture); consequently, cells from this experiment were excluded from the analyses in Fig. 5. The method by Li et al. [21] was used to compute Ka/Ks statistic controlling for the mutational spectra. The null distribution was built by 3000 permutations controlling the respective mutational spectrum in either cell type.

### Alignment quality assessment for linkage analysis

During analyzing SNV linkages, some significantly linked SNVs were observed in a small fraction of amplicons and showed suboptimal read quality. For example, 6 variants around 13775 were located on reads starting ~ 80 bp downstream the 5′ end of the PCR forward primer (Additional file 39: Figure S25) and appeared as linked SNVs. To avoid suboptimal alignment that could be a major confounder for this analysis, a stricter set of filtering criteria was used: (1) reads must be on the positive strand only, (2) reads must start off-5′ end only by + / − 1 bp, (3) read length must be ≥ 135 bp. Using this stricter processing, 776 total SNV sites and 209 SNV sites shared by ≥ 3 mitochondria were observed. Comparing with the permissive processing (Additional file 40: Figure S26), 92.6% (776) SNV sites survived the stricter filtering (Additional file 40: Figure S26A). The stricter filtering also led to 22 new SNV sites shared by ≥ 3 mitochondria because of an increase in the VAF after the stricter filtering (Additional file 40: Figure S26B).

### SNV linkage analysis

To detect linked SNVs, the SNV sites that had non-missing allele calls in ≥ 10 mitochondria and that had ≥ 5% mitochondria with the minor allele were filtered. Furthermore, cells with ≥ 20 mitochondria were filtered to study linkage at the cell level. The association between a pair of SNVs was quantified by a one-tail Fisher’s exact test. Briefly, for each cell and for each pair of the filtered SNV sites, whether the observed odds ratio of any combination of observed alleles significantly exceeded the random was tested. The *p*-values were subsequently corrected for multiple tests using the Benjamini–Hochberg method [87].

### Statistics

Statistical analyses and graphics are done with R Project for Statistical Computing (RRID:SCR_001905), circlize (RRID:SCR_002141), and ggplot2 (RRID:SCR_014601).

### Supplementary Information

Additional file 1: Figure S1. An overview of the distribution of the total SNVs. (A) The distribution of the number of mitochondria captured per cell per mouse. The mitochondria number isolated per neuron or astrocyte is depicted as a blue or an orange dot respectively. (B) The graph depicts a SNVs’ presence (as red dot) in each mitochondrion sample on the y-axis (each row is one individual mitochondrion sample) across the mt-genome (x-axis). The y axis on the right shows identity of each mitochondrion sample with respect to the mouse, cell type, cell, library ID barcode. (C) The number of SNVs is represented on the y-axis in each target region genes depicted on the x-axis. SNVs color coded by the number of shared mitochondria, cells, and mice.

Additional file 2: Dataset S1. Sample metadata for SMITO dataset.

Additional file 3: Figure S2. Distribution of the SNV counts in the SMITO dataset. Histograms showing the distribution of the SNV count in each mitochondrion for each of the 102 cells analyzed. Each histogram represents a cell (102 total) and is labelled with the cell type and the animal ID.

Additional file 4: Figure S3. Mutational Spectra for the mt-genome. (A) Intrinsic C <  > T, G <  > A tendency due to mt-genome base composition and (B) the SMITO mutational spectra accounting for the intrinsic tendency.

Additional file 5: Dataset S2. SNV Impact Annotation for 1032 SNVs from SMITO dataset.

Additional file 6: Figure S4. Comparison of Cells and Mitochondria Sharing Deleterious and Tolerated Nonsynonymous SNVs. (A) Number of cells or (B) mitochondria sharing the nonsynonymous SNVs, which were categorized as “deleterious” and “tolerated” by SIFT (for high-confidence only). The *p*-value is from Wilcoxon’s rank-sum test.

Additional file 7: Figure S5. Illustration of the anti-codon base changes in a tRNA SNV 9423:C > T.

Additional file 8: Dataset S3. SMITO dataset Comparison with mouse strains data.

Additional file 9: Figure S6. The distribution of the VAF and number of SNVs shared by the single-mt samples. (A) The relationship between the average AF (y-axis) and the number of cells sharing the SNVs. (B) The relationship between the average AF (y-axis) and the number of mitochondria sharing the SNVs. (C) The number of SNVs (y-axis) stratified by the number of cells sharing these SNVs. (D) The number of SNVs (y-axis) stratified by the number of shared mitochondria with these SNVs.

Additional file 10: Figure S7. Comparison between the inherited and the somatic SNVs in mutational propensity. (A) Inherited SNVs’ per-base variant rate on the y-axis in each target gene loci on the x-axis. (B) Somatic SNVs’ per-base variant rate on the y-axis in each target gene locus on the x-axis. (C) The Ti/Tv ratio of inherited (red) and somatic (blue) SNVs for each target region gene. (D) The Ti/Tv ratio of inherited (red) and somatic (blue) SNVs for each group namely rRNA, tRNA, synonymous, nonsynonymous, D-loop, intergenic and the overall average). In (C-D), highest bars were capped due to infinite ratios (no transversion).

Additional file 11: Table S1. List of the genome accession numbers of the mouse strains, species and populations.

Additional file 12: Figure S8. Between-genera, species, strains and population data comparison. Neighbor-joining phylogenetic trees of (A) 8 Mus species and (B) two Mus musculus populations. Ti/Tv ratios for segregating sites in (C) whole mt-genome and (D) regions assayed by SMITO between genera (Mus musculus and Rattus norvegicus), Mus species, Mus musculus strains, and Mus musculus populations in respective functional class. In panel D, the highest bars were capped due to being infinite (i.e. transitions only).

Additional file 13: Dataset S4. Ti/Tv ratio comparisons for genera, species, strains and population data.

Additional file 14: Dataset S5. MK-test results for population data (castaneus-vs-domesticus).

Additional file 15: Figure S9. Comparing inherited SNVs’ AF variation across the mouse, cell, and mitochondrion level. (A) The observed (the colored line) and the null (gray lines) distribution of the AF variation proportion at the mouse (left), cell (middle) and mitochondrion (right) level. (B) The mitochondrion-level (top), cell-level (bottom left) and mouse-level (bottom right) AF distribution of 9027: G > A. The color indicates the mouse identity.

Additional file 16: Figure S10. Uniform Manifold Approximation and Projection (UMAP) graph across all single-mitochondria samples. Left, UMAP embedding of the single mitochondria color-coded by the mouse. Right, UMAP embedding of the single mitochondria color-coded by the cell.

Additional file 17:Dataset S6. Cell-type comparison of VAF variance in neurons and astrocytes.

Additional file 18: Figure S11. RNAfold predicted tRNA secondary structure changes caused by 9419:C > T. (A) The secondary structure of mt-Tg with the reference allele. (B) The secondary structure of mt-Tg with the variant allele.

Additional file 19: Figure S12. Ka/Ks statistics in all SMITO SNVs. The background distribution was derived from simulated random mutations (3000 times permutation) conditioned on the overall mutational spectrum. The red dashed-line here represents the observed Ka/Ks from total SNVs.

Additional file 20: Dataset S7. Ks/Ks statistic data for astrocytes and neurons.

Additional file 21: Figure S13. IGV snapshots showing the 6430–6432 linkage on the same haplotype. (A) An example of mitochondrion carrying the linked SNVs in the same reads in Mouse10. (B) An example of mitochondrion carrying the linked SNVs in the same reads in Mouse12.

Additional file 22: Figure S14. Representative images of primary mouse neurons and astrocytes. A representative image of a (A) pyramidal, (B) bipolar and (C) unipolar neuron, shown by blue arrows and (D-F) astrocytes shown by orange arrows. Scale bars are 20 microns.

Additional file 23: Figure S15. Single mitochondrion isolation from individual neuron or astrocyte cells based on Poisson statistics. (A) Flow cytometry characterization of antibody coated microbeads. (B) Gating strategy for isolating single mitochondrion from cell lysate of individual neuron or astrocyte staining by MitoTracker Red. Red dots indicated microbeads-only control, blue dots indicate microbeads captured unstained mitochondria as negative control. (C) MitoTracker gating on microbeads incubated with mitochondria stained by MitoTracker Red as positive control. (D) Comparing the MitoTracker signal from samples in B and C. Red plot indicate microbeads captured MitoTracker stained mitochondria. The orange plot depicts microbeads-only control. Blue plot depicts microbeads captured unstained mitochondria. (E) Experimental and theoretical analysis on single mt capture based on Poisson statistics. x-axis is the ratio of input mitochondria number to input microbead number. Y-axis is the probability of microbeads that captured a mitochondrion. Error bars indicate standard deviation from *n* = 3 independent experiments.

Additional file 24: Figure S16. RCA products from single mitochondrion samples. Shown here is a representative ethidium bromide agarose gel electrophoresis with RCA products. Each well in (A) and (B) were loaded with 25-fold diluted RCA products from each mitochondrion except the control wells E5-E12 in (B). E5-6 are negative controls, E7-8 are positive controls (0.1 picogram isolated mtDNA from mouse brain used as a template), E9-10 are random primer control and E11-12 are no-template controls.

Additional file 25: Table S2. List of the sequences of the 10 barcodes (M1-10) for mitochondria multiplexing.

Additional file 26:Table S3. List of the sequences of the forward/reverse PCR primers for selected specific target regions.

Additional file 27: Table S4. Edit distances between any pair of the mitochondrial-barcodes.

Additional file 28: Figure S17. Sample size and quality assessment for each target region. The heatmap showing the number of bases with sufficient depth (≥ 50) in each mt (row) for each PCR target region (depicted as columns).

Additional file 29: Figure S18. Per-base read depth for each mt-barcode and each PCR target region. Each subfigure depicts one of the 120 combinations of mt-barcode and PCR target region, and an individual curve (and color) within a subfigure represents a single mitochondrion.

Additional file 30: Figure S19. Phred score threshold choice and its impact on read coverage. (A) Scatter plot showing the theoretical error rate (Phred score, x-axis) and the observed mismatch rate (y-axis). (B) Read coverage in target Region3 before (top) and after (bottom) Q30 filtering.

Additional file 31: Dataset S8. SNV AF table and samples support for each of the 1032 SNVs.

Additional file 32: Figure S20. STAR-BWA pipeline comparison in SNV location, depth and VAF. (A) Venn diagram showing the overlap between STAR and BWA in all SNV sites. (B) Venn diagram showing the overlap between STAR and BWA in SNV sites shared by at least three mice. (C) Scatter plot showing the depth of STAR (x-axis) and BWA (y-axis). (D) Scatter plot showing the VAF of STAR (x-axis) and BWA (y-axis).

Additional file 33: Figure S21. Boxplots of the read depth in each of the 12 PCR target regions. The average read depth (log 10) for single-mt samples [single-mtDNA ( +) primer ( +)] and positive [pooled mtDNA ( +) primer ( +)] or negative controls [mtDNA (-) RCA (-)] or other controls [mtDNA ( +) primer (-), mtDNA (-) primer ( +)] in Region 1 – 12.

Additional file 34: Dataset S9. QC statistics from pipeline preprocessing.

Additional file 35: Figure S22. Correction for Soft-clipping by STAR on 9027:G > A. The VAF difference between before and after patching the non-reference base As that were soft-clipped by STAR. (A) Before the correction. (B) After the correction.

Additional file 36: Figure S23. Mouse NUMTs Analysis on the SMITO dataset. (A) Homologous mtDNA shown as links; colors denote individual chromosomes. (B) Representative example of D5000 Agilent ScreenTape with PCR product from targeting single mt RCA product for a mt region (SNV6) and 2 independent nuclear regions (Pet-1 and Gapdh).

Additional file 37: Dataset S10. Mouse NUMTs Analysis on SMITO data

Additional file 38: Figure S24. Sequence comparison between UCSC GRCm38 (C57BL/6 J) and C57BL/6NJ from the Mouse Genomes Project in the region highlighting two differences.

Additional file 39: Figure S25. Examples of suboptimal alignments and the mutations in linkage. The green box at the top highlights the optimal alignments, while the three red boxes represent suboptimal cases.

Additional file 40: Figure S26. Comparison between the permissive and the stricter filtering. (A) Venn diagram showing the overlap of all SNV sites between the permissive and the stricter filtering. (B) Venn diagram showing the overlap of the SNV sites shared by at least 3 mitochondria between the permissive and the stricter filtering.

## Data Availability

All data generated or analyzed during this study are included in this published article, its supplementary information files and publicly available repositories. The raw sequencing reads, the metadata and the processed AF table have been deposited to GEO under the accession number GSE218122: https://www.ncbi.nlm.nih.gov/geo/query/acc.cgi?acc=GSE218122. Novel experimental reagents are available upon request. The SMITO SNV Pipeline is now citable by the DOI: 10.5281/zenodo.12210095. The Data Analysis Script is citable by the DOI: 10.5281/zenodo.12210103.

## Abbreviations

mt: Mitochondrial
SNV: Single-nucleotide variant
AF: Allele frequency
VAF: Variant allele frequency
RCA: Rolling circle amplification
SMITO: Single-mitochondria dataset
SIFT: Sorting Intolerant from Tolerant
Ti: Transitions
Tv: Transversions
MS: Mean-of-squares
Ka: Number of nonsynonymous substitutions per nonsynonymous site
Ks: Number of synonymous substitutions per synonymous site
OXPHOS: Oxidative phosphorylation
ROS: Reactive oxygen species
NGS: Next-Generation Sequencing
NUMTs: Nuclear-embedded mitochondrial DNA Sequences
