# Supplementary figures and images for: Single-mitochondrion sequencing uncovers distinct mutational patterns and heteroplasmy landscape in mouse astrocytes and neurons
Source: BMC Biol. 2024 Jul 29;22:162. doi: 10.1186/s12915-024-01953-7 (PMC11287894; doi:10.1186/s12915-024-01953-7)

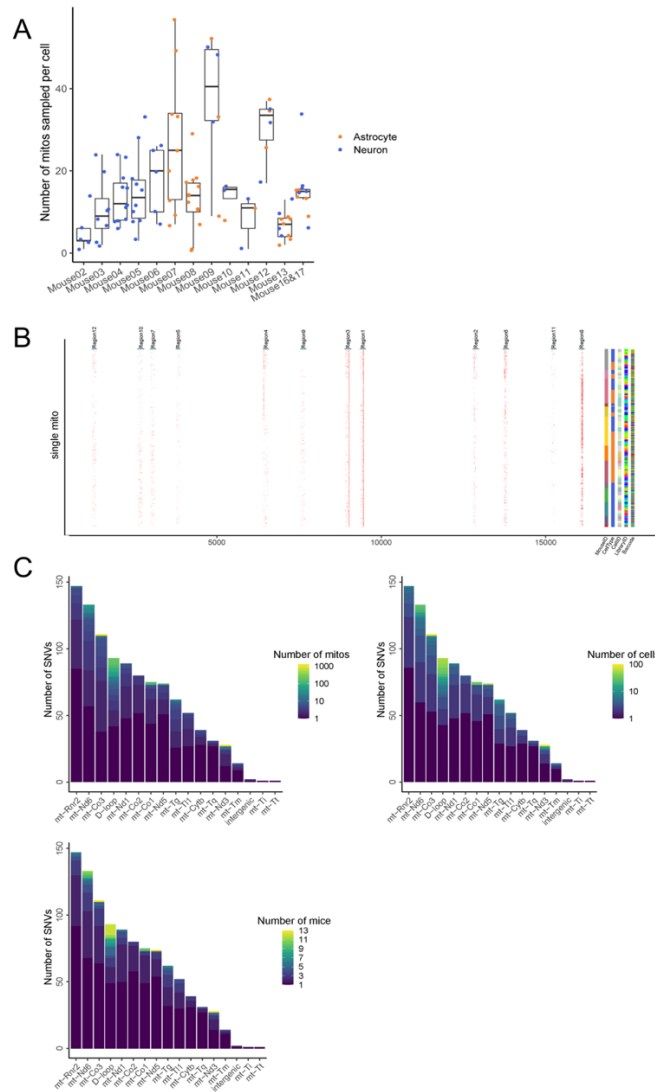

**Figure S1. An overview of the distribution of the total SNVs.**

Supplement: Supplementary file 1 — Additional file 1: Figure S1. An overview of the distribution of the total SNVs. (A) The distribution of the number of mitochondria captured per cell per mouse. The mitochondria number isolated per neuron or astrocyte is depicted as a blue or an orange dot respectively. (B) The graph depicts a SNVs’ presence (as red dot) in each mitochondrion sample on the y-axis (each row is one individual mitochondrion sample) across the mt-genome (x-axis). The y axis on the right shows identity of each mitochondrion sample with respect to the mouse, cell type, cell, library ID barcode. (C) The number of SNVs is represented on the y-axis in each target region genes depicted on the x-axis. SNVs color coded by the number of shared mitochondria, cells, and mice. [file 12915_2024_1953_MOESM1_ESM.pdf]

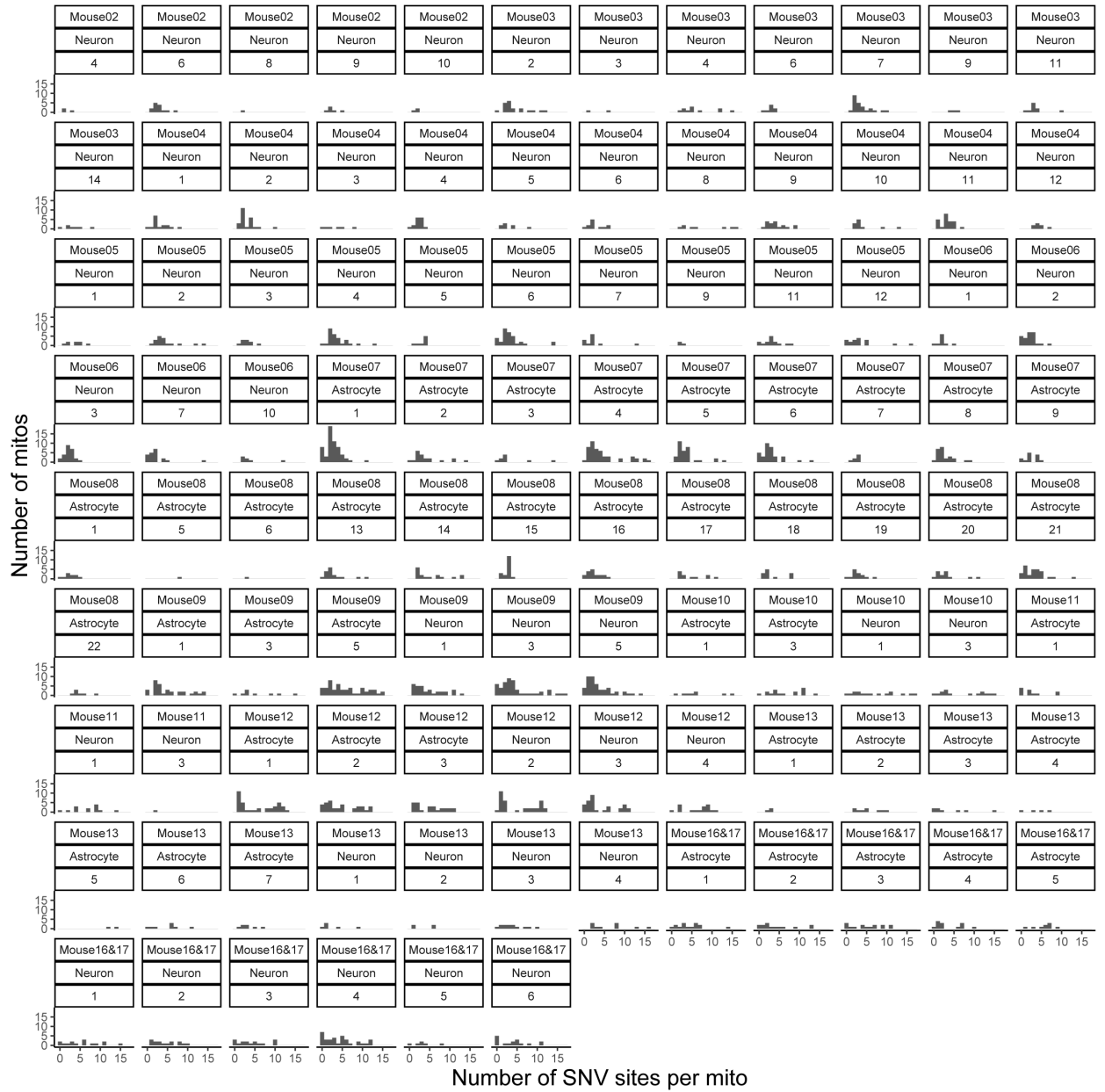

**Figure S2. Distribution of the SNV counts in the SMITO dataset.**

Supplement: Supplementary file 3 — Additional file 3: Figure S2. Distribution of the SNV counts in the SMITO dataset. Histograms showing the distribution of the SNV count in each mitochondrion for each of the 102 cells analyzed. Each histogram represents a cell (102 total) and is labelled with the cell type and the animal ID. [file 12915_2024_1953_MOESM3_ESM.pdf]

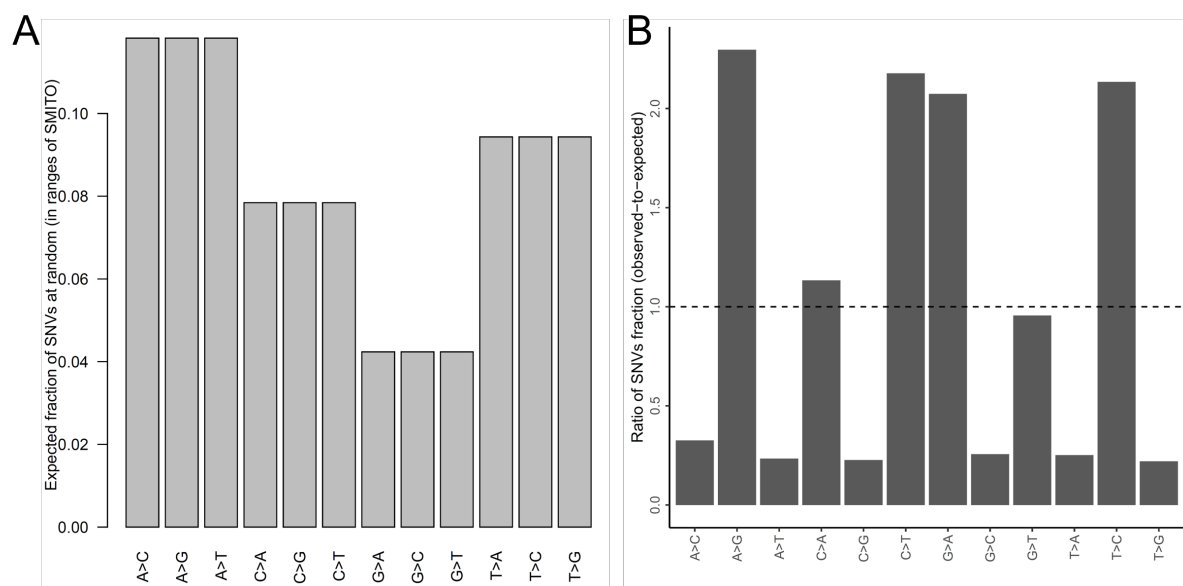

**Figure S3. Mutational Spectra for the mt-genome.**

Supplement: Supplementary file 4 — Additional file 4: Figure S3. Mutational Spectra for the mt-genome. (A) Intrinsic C < > T, G < > A tendency due to mt-genome base composition and (B) the SMITO mutational spectra accounting for the intrinsic tendency. [file 12915_2024_1953_MOESM4_ESM.pdf]

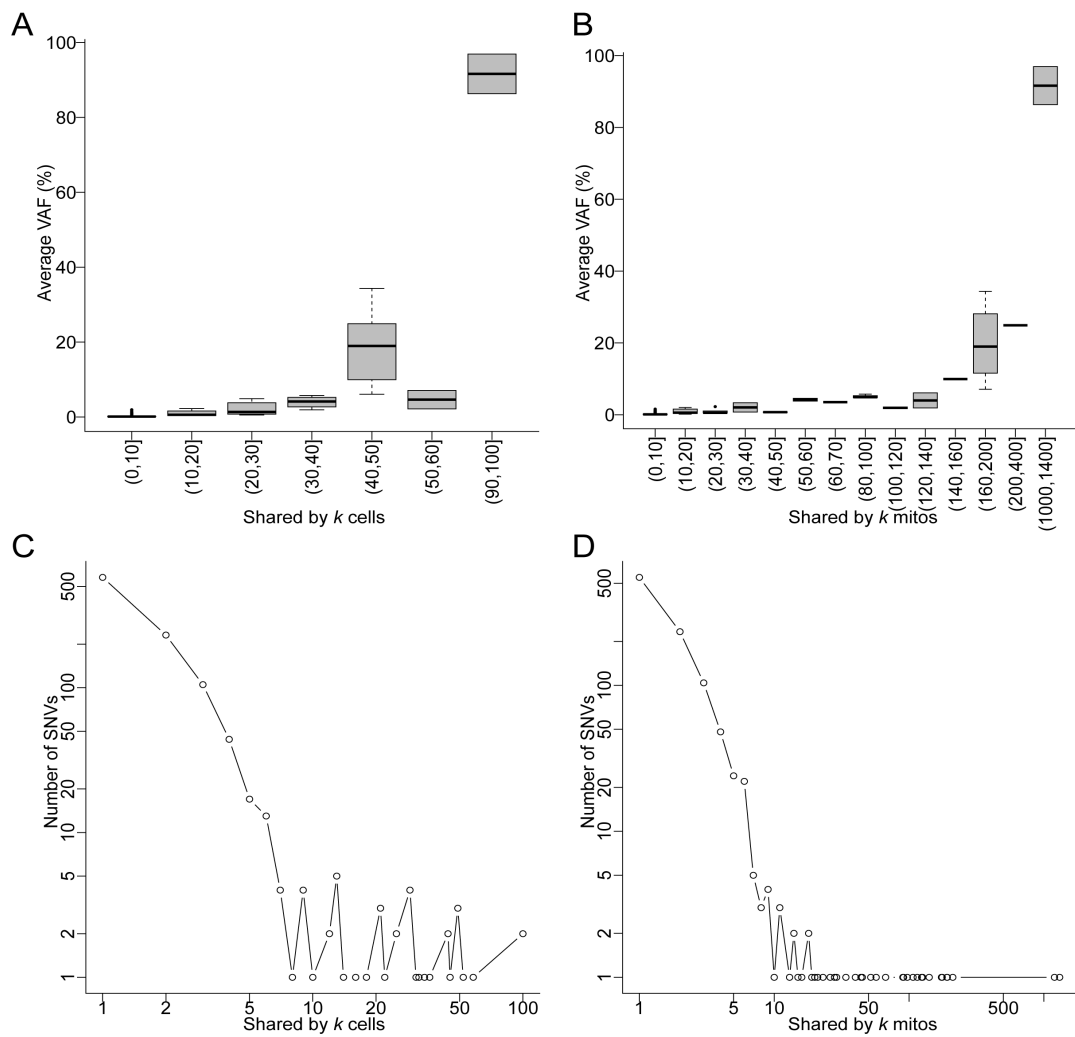

**Figure S6. The distribution of the VAF and number of SNVs shared by the single-mt samples.**

Supplement: Supplementary file 9 — Additional file 9: Figure S6. The distribution of the VAF and number of SNVs shared by the single-mt samples. (A) The relationship between the average AF (y-axis) and the number of cells sharing the SNVs. (B) The relationship between the average AF (y-axis) and the number of mitochondria sharing the SNVs. (C) The number of SNVs (y-axis) stratified by the number of cells sharing these SNVs. (D) The number of SNVs (y-axis) stratified by the number of shared mitochondria with these SNVs. [file 12915_2024_1953_MOESM9_ESM.pdf]

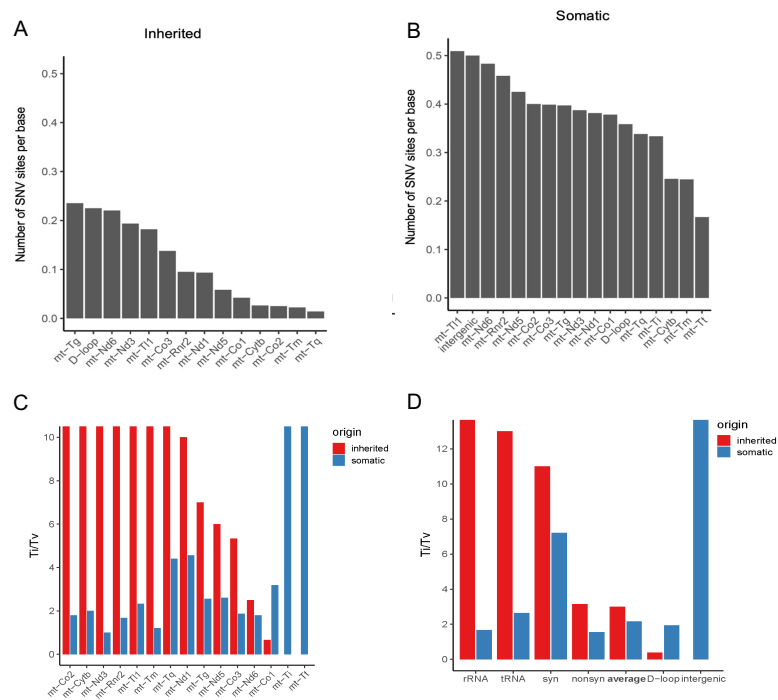

**Figure S7. Comparison between the inherited and the somatic SNVs in mutational propensity.**

Supplement: Supplementary file 10 — Additional file 10: Figure S7. Comparison between the inherited and the somatic SNVs in mutational propensity. (A) Inherited SNVs’ per-base variant rate on the y-axis in each target gene loci on the x-axis. (B) Somatic SNVs’ per-base variant rate on the y-axis in each target gene locus on the x-axis. (C) The Ti/Tv ratio of inherited (red) and somatic (blue) SNVs for each target region gene. (D) The Ti/Tv ratio of inherited (red) and somatic (blue) SNVs for each group namely rRNA, tRNA, synonymous, nonsynonymous, D-loop, intergenic and the overall average). In (C-D), highest bars were capped due to infinite ratios (no transversion). [file 12915_2024_1953_MOESM10_ESM.pdf]

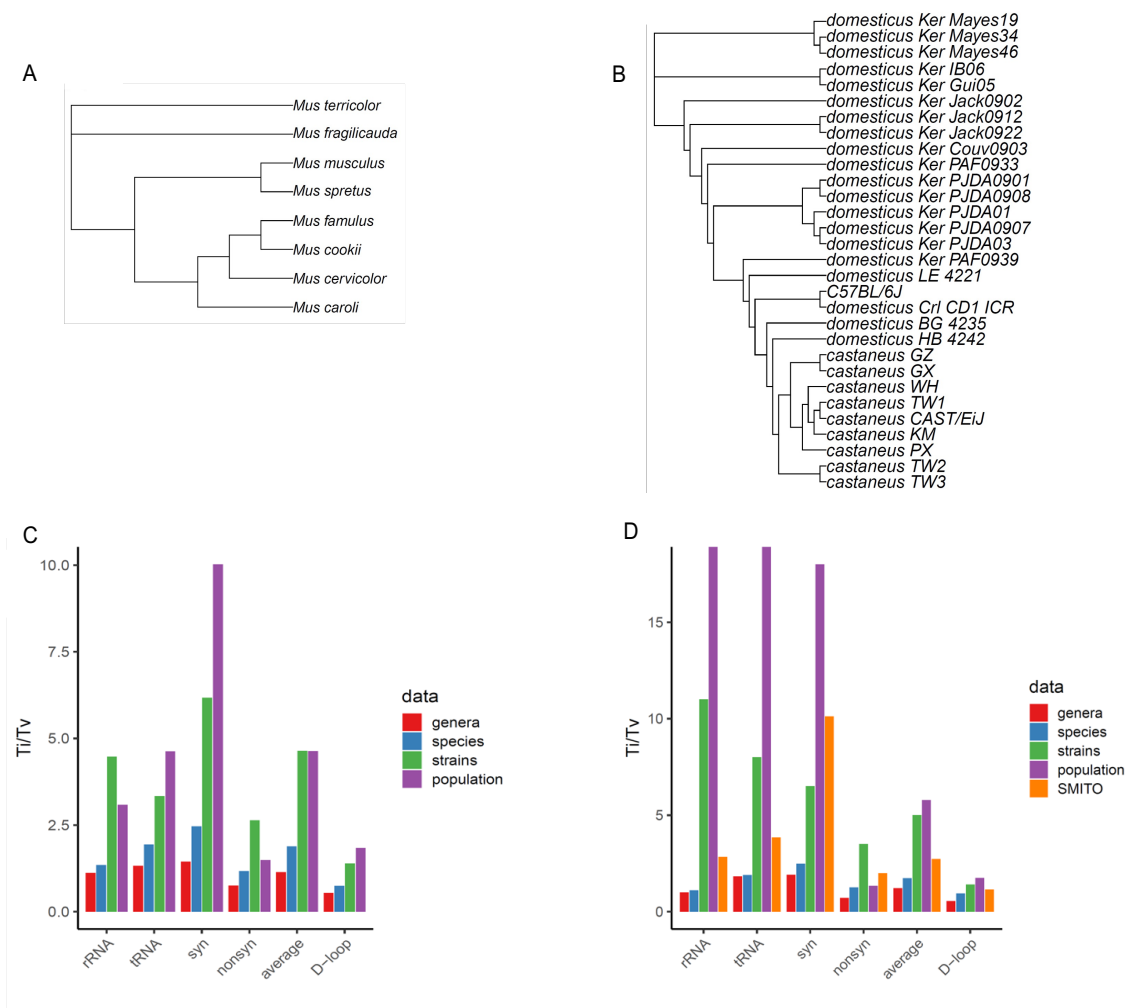

**Figure S8. Between-genera, species, strains and population data comparison.**

Supplement: Supplementary file 12 — Additional file 12: Figure S8. Between-genera, species, strains and population data comparison. Neighbor-joining phylogenetic trees of (A) 8 Mus species and (B) two Mus musculus populations. Ti/Tv ratios for segregating sites in (C) whole mt-genome and (D) regions assayed by SMITO between genera (Mus musculus and Rattus norvegicus), Mus species, Mus musculus strains, and Mus musculus populations in respective functional class. In panel D, the highest bars were capped due to being infinite (i.e. transitions only). [file 12915_2024_1953_MOESM12_ESM.pdf]

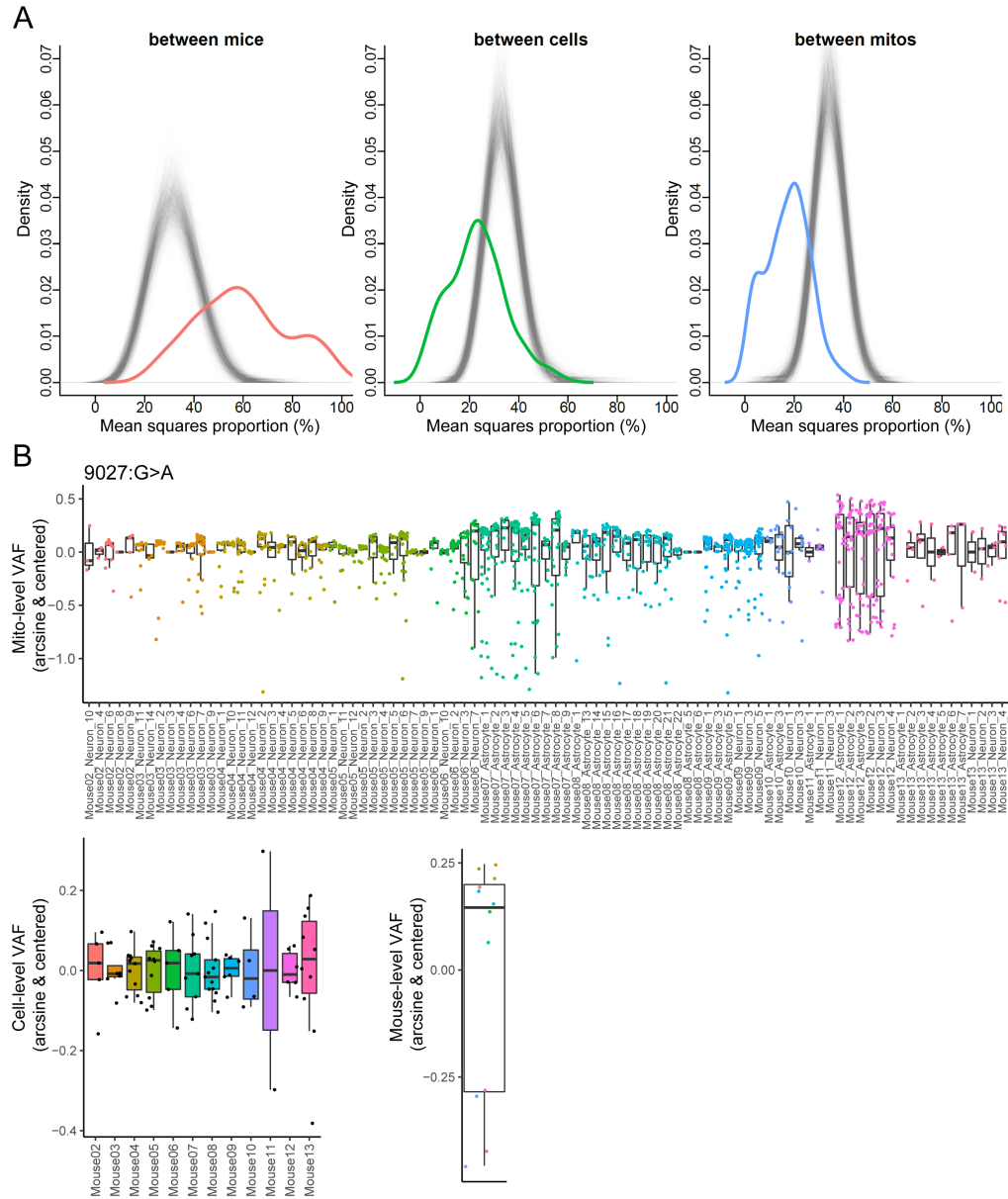

**Figure S9. Comparing inherited SNVs' AF variation across the mouse, cell, and mitochondrion level.**

Supplement: Supplementary file 15 — Additional file 15: Figure S9. Comparing inherited SNVs’ AF variation across the mouse, cell, and mitochondrion level. (A) The observed (the colored line) and the null (gray lines) distribution of the AF variation proportion at the mouse (left), cell (middle) and mitochondrion (right) level. (B) The mitochondrion-level (top), cell-level (bottom left) and mouse-level (bottom right) AF distribution of 9027: G > A. The color indicates the mouse identity. [file 12915_2024_1953_MOESM15_ESM.pdf]

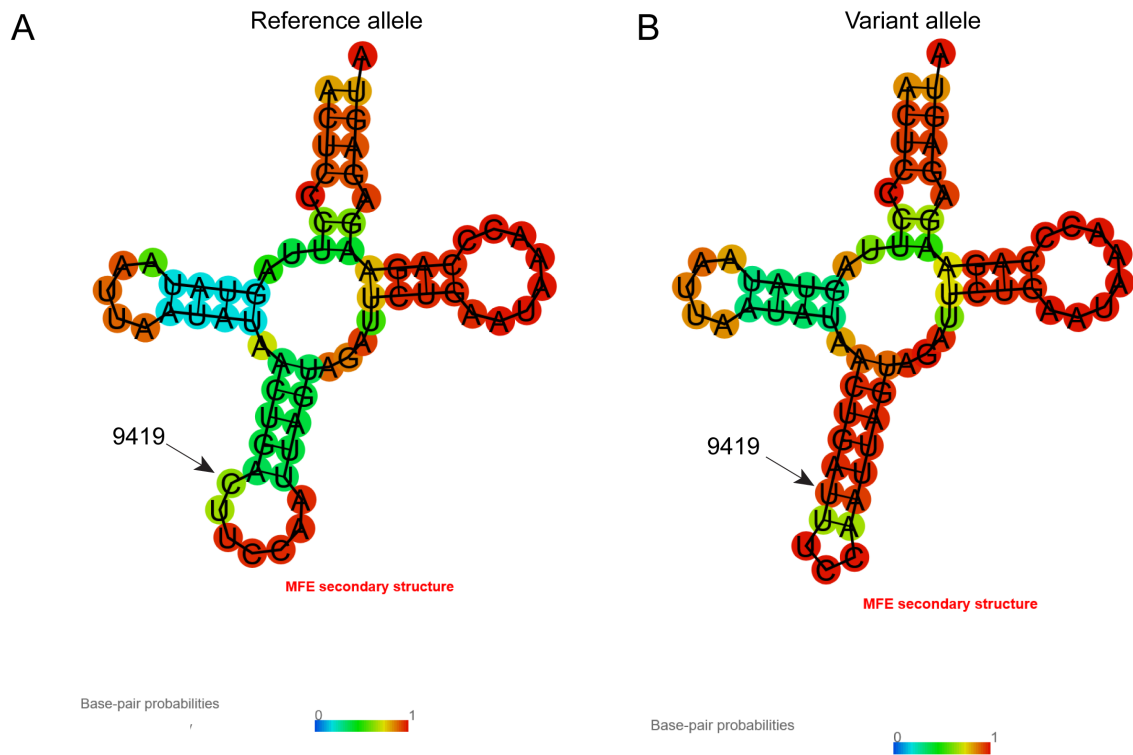

**Figure S11. RNAfold predicted tRNA secondary structure changes caused by 9419:C>T.**

Supplement: Supplementary file 18 — Additional file 18: Figure S11. RNAfold predicted tRNA secondary structure changes caused by 9419:C > T. (A) The secondary structure of mt-Tg with the reference allele. (B) The secondary structure of mt-Tg with the variant allele. [file 12915_2024_1953_MOESM18_ESM.pdf]

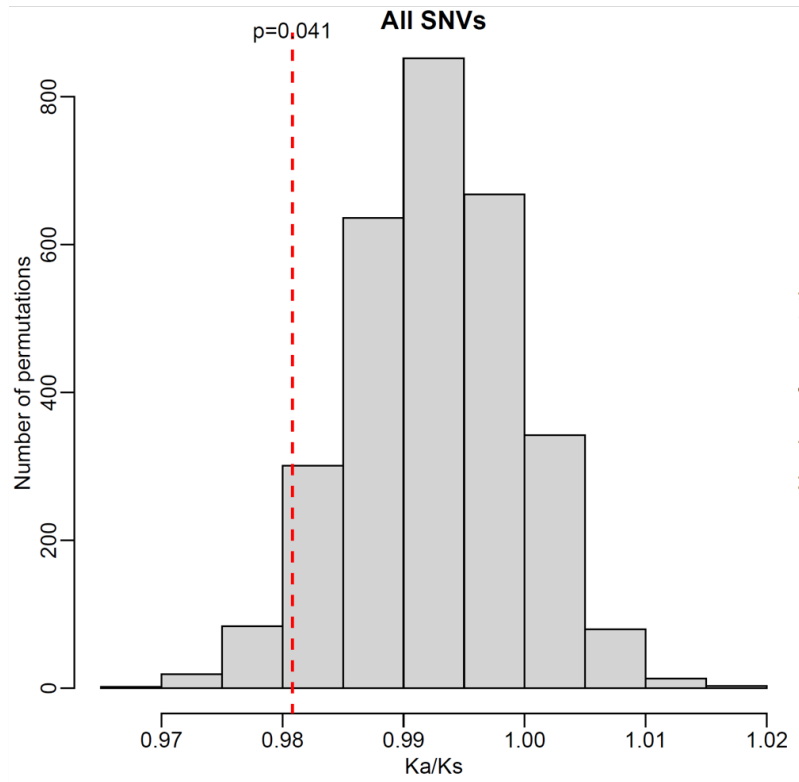

**Figure S12. Ka/Ks statistics in all SMITO SNVs.**

Supplement: Supplementary file 19 — Additional file 19: Figure S12. Ka/Ks statistics in all SMITO SNVs. The background distribution was derived from simulated random mutations (3000 times permutation) conditioned on the overall mutational spectrum. The red dashed-line here represents the observed Ka/Ks from total SNVs. [file 12915_2024_1953_MOESM19_ESM.pdf]

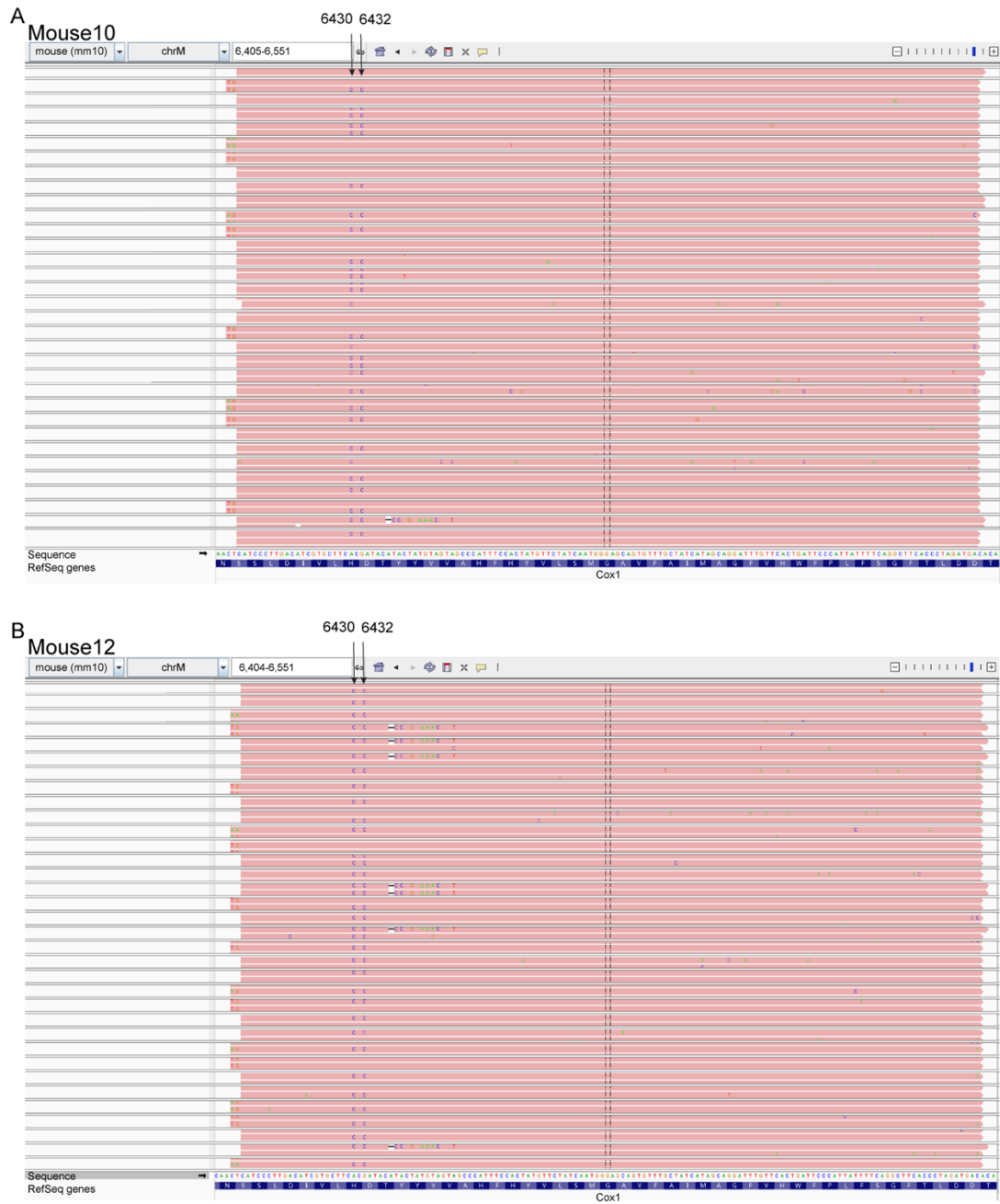

**Figure S13. IGV snapshots showing the 6430-6432 linkage on the same haplotype.**

Supplement: Supplementary file 21 — Additional file 21: Figure S13. IGV snapshots showing the 6430–6432 linkage on the same haplotype. (A) An example of mitochondrion carrying the linked SNVs in the same reads in Mouse10. (B) An example of mitochondrion carrying the linked SNVs in the same reads in Mouse12. [file 12915_2024_1953_MOESM21_ESM.pdf]

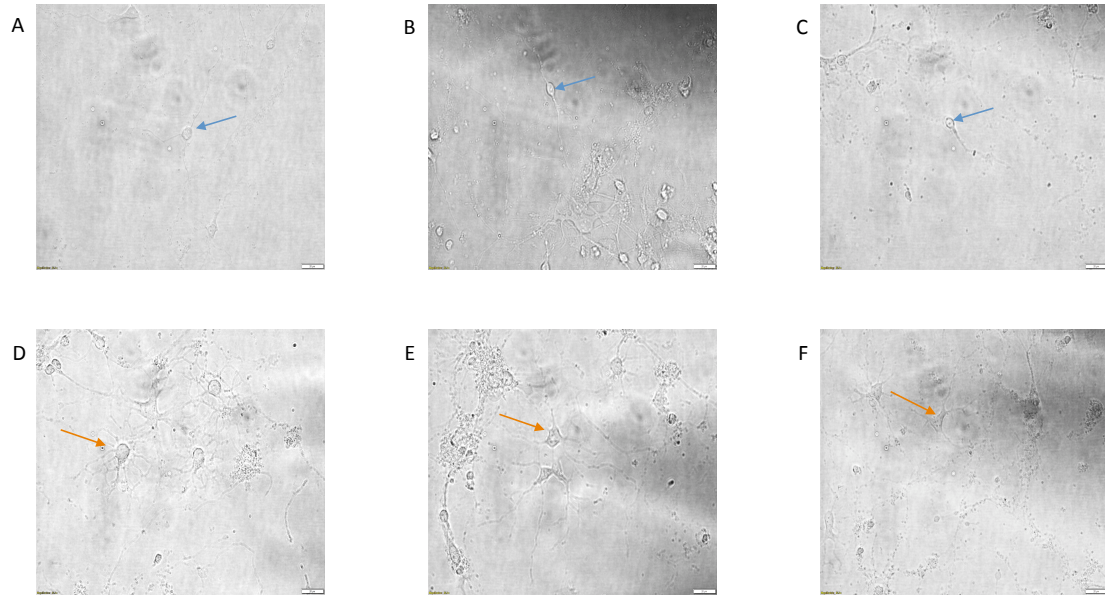

**Figure S14. Representative images of primary mouse neurons and astrocytes.**

Supplement: Supplementary file 22 — Additional file 22: Figure S14. Representative images of primary mouse neurons and astrocytes. A representative image of a (A) pyramidal, (B) bipolar and (C) unipolar neuron, shown by blue arrows and (D-F) astrocytes shown by orange arrows. Scale bars are 20 microns. [file 12915_2024_1953_MOESM22_ESM.pdf]

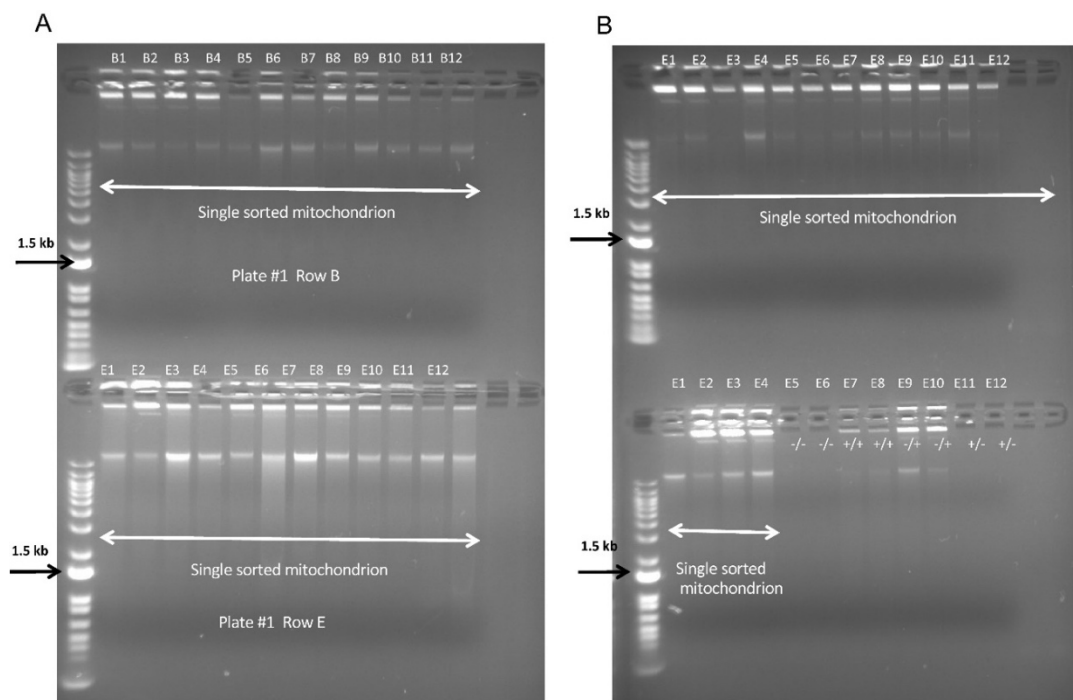

**Figure S16. RCA products from single mitochondrion samples.**

Supplement: Supplementary file 24 — Additional file 24: Figure S16. RCA products from single mitochondrion samples. Shown here is a representative ethidium bromide agarose gel electrophoresis with RCA products. Each well in (A) and (B) were loaded with 25-fold diluted RCA products from each mitochondrion except the control wells E5-E12 in (B). E5-6 are negative controls, E7-8 are positive controls (0.1 picogram isolated mtDNA from mouse brain used as a template), E9-10 are random primer control and E11-12 are no-template controls. [file 12915_2024_1953_MOESM24_ESM.pdf]

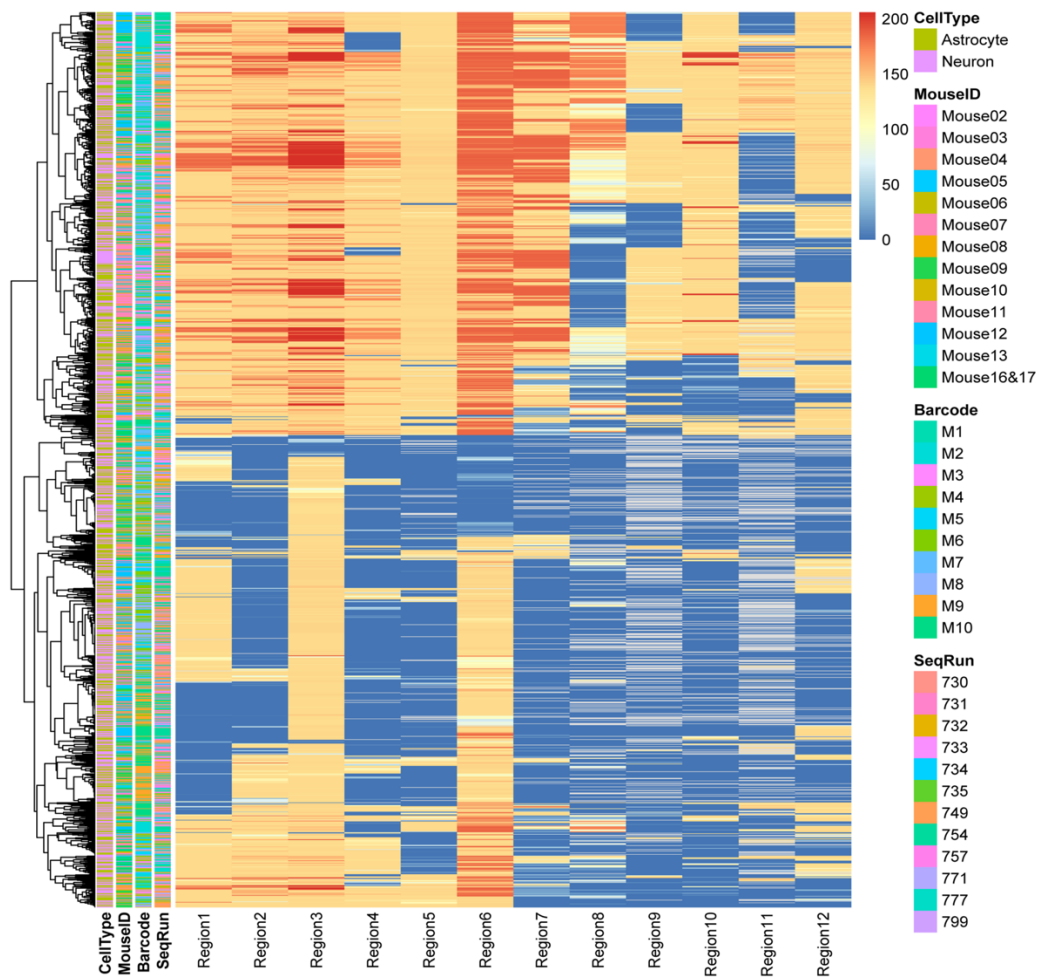

**Figure S17. Sample size and quality assessment for each target region.**

Supplement: Supplementary file 28 — Additional file 28: Figure S17. Sample size and quality assessment for each target region. The heatmap showing the number of bases with sufficient depth (≥ 50) in each mt (row) for each PCR target region (depicted as columns). [file 12915_2024_1953_MOESM28_ESM.pdf]

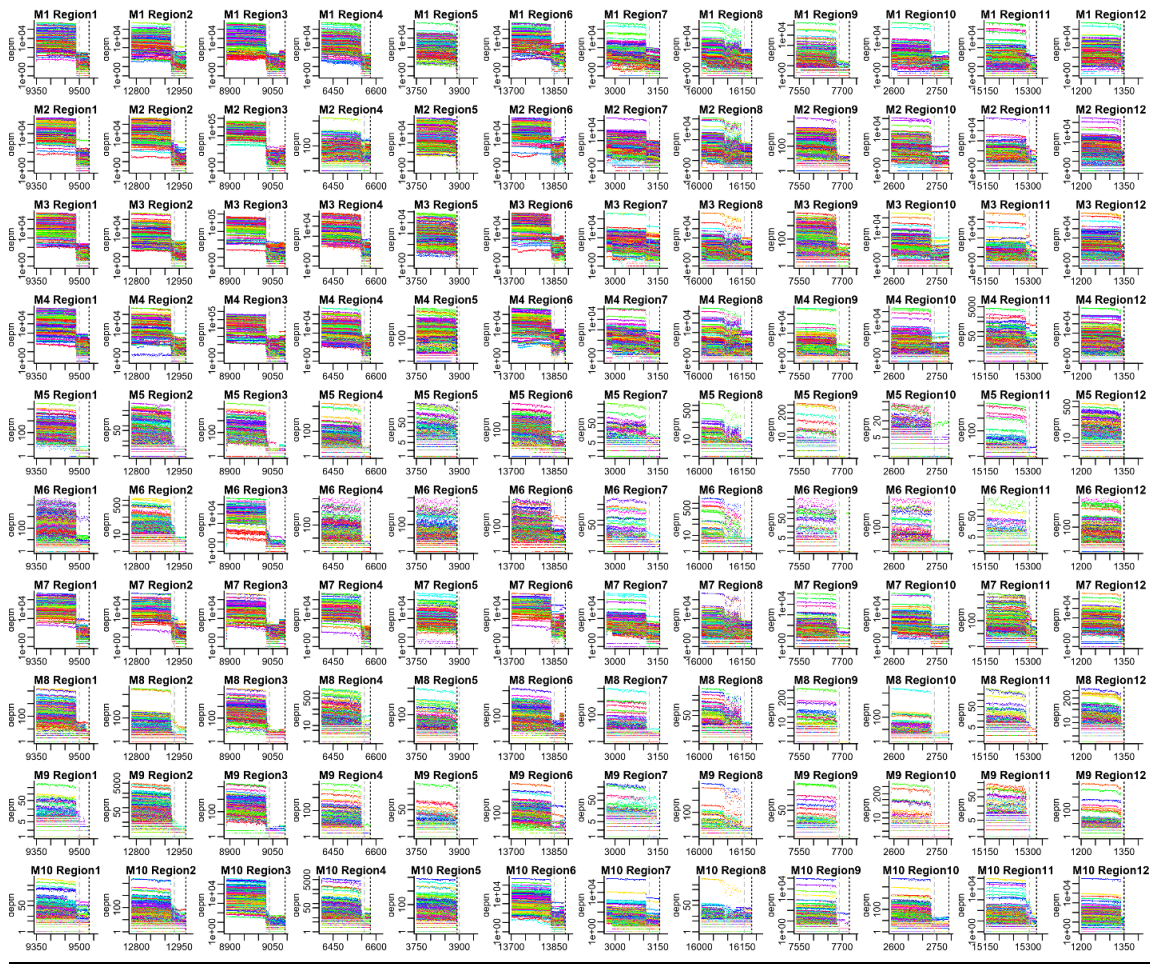

**Figure S18. Per-base read depth for each mt-barcode and each PCR target region.**

Supplement: Supplementary file 29 — Additional file 29: Figure S18. Per-base read depth for each mt-barcode and each PCR target region. Each subfigure depicts one of the 120 combinations of mt-barcode and PCR target region, and an individual curve (and color) within a subfigure represents a single mitochondrion. [file 12915_2024_1953_MOESM29_ESM.pdf]

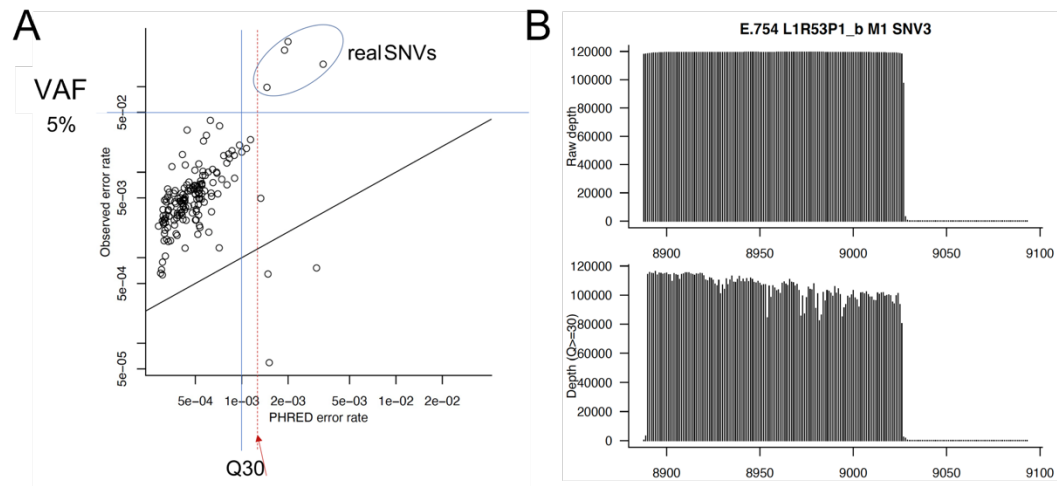

**Figure S19. Phred score threshold choice and its impact on read coverage.**

Supplement: Supplementary file 30 — Additional file 30: Figure S19. Phred score threshold choice and its impact on read coverage. (A) Scatter plot showing the theoretical error rate (Phred score, x-axis) and the observed mismatch rate (y-axis). (B) Read coverage in target Region3 before (top) and after (bottom) Q30 filtering. [file 12915_2024_1953_MOESM30_ESM.pdf]

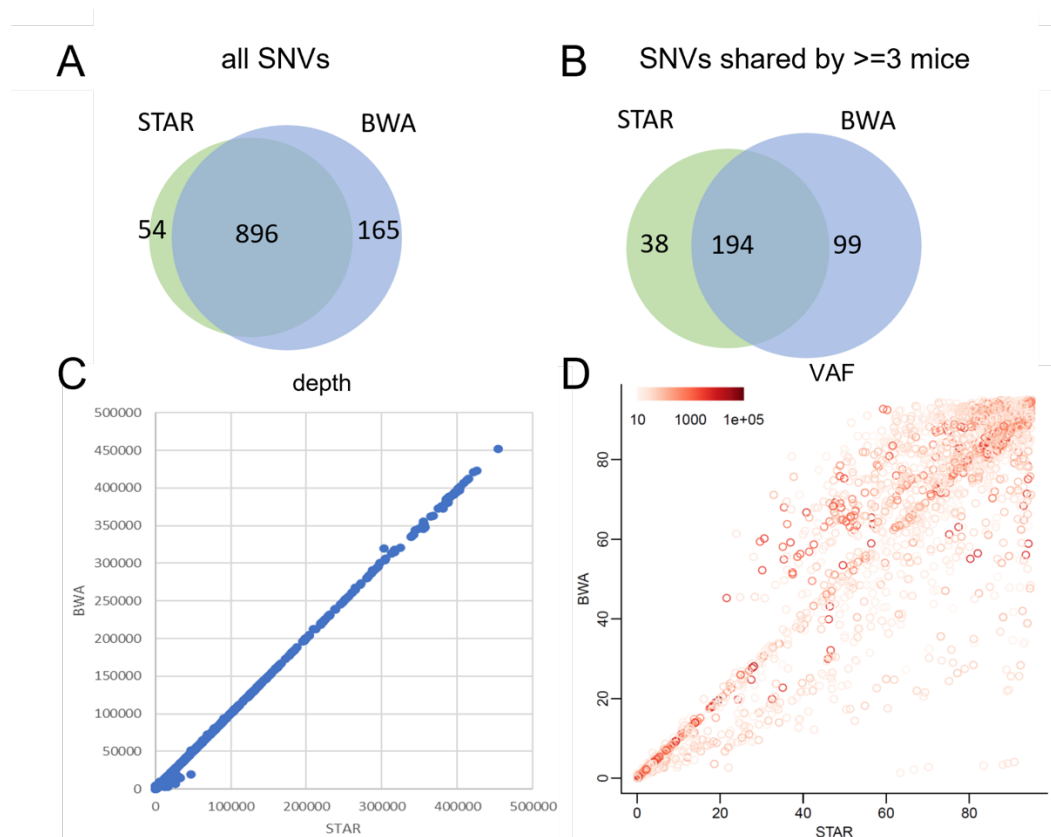

**Figure S20. STAR-BWA pipeline comparison in SNV location, depth and VAF.**

Supplement: Supplementary file 32 — Additional file 32: Figure S20. STAR-BWA pipeline comparison in SNV location, depth and VAF. (A) Venn diagram showing the overlap between STAR and BWA in all SNV sites. (B) Venn diagram showing the overlap between STAR and BWA in SNV sites shared by at least three mice. (C) Scatter plot showing the depth of STAR (x-axis) and BWA (y-axis). (D) Scatter plot showing the VAF of STAR (x-axis) and BWA (y-axis). [file 12915_2024_1953_MOESM32_ESM.pdf]

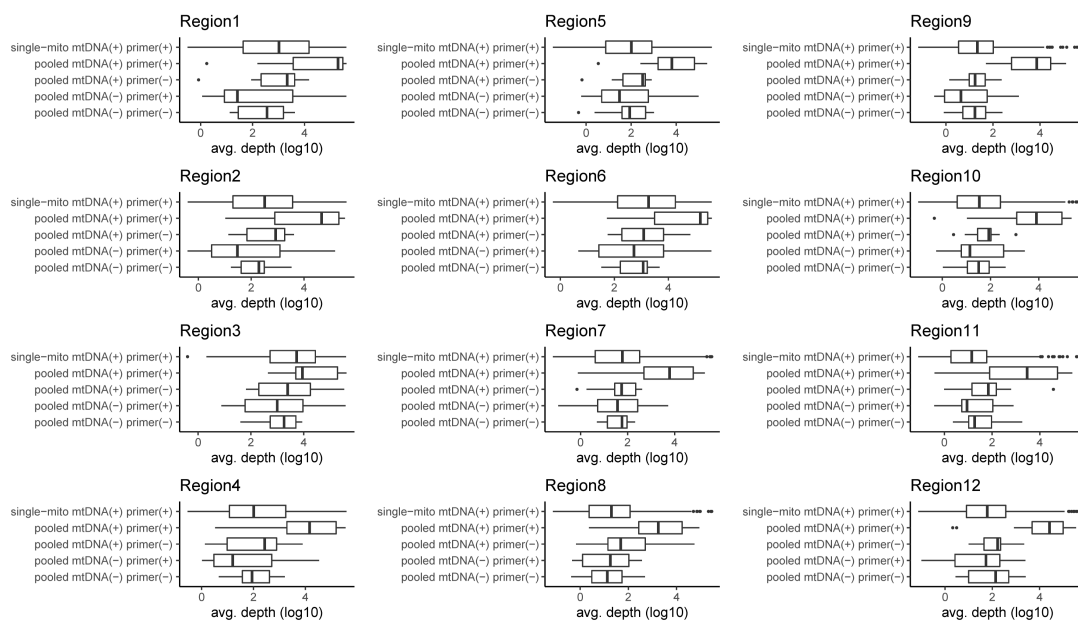

**Figure S21. Boxplots of the read depth in each of the 12 PCR target regions.**

Supplement: Supplementary file 33 — Additional file 33: Figure S21. Boxplots of the read depth in each of the 12 PCR target regions. The average read depth (log 10) for single-mt samples [single-mtDNA ( +) primer ( +)] and positive [pooled mtDNA ( +) primer ( +)] or negative controls [mtDNA (-) RCA (-)] or other controls [mtDNA ( +) primer (-), mtDNA (-) primer ( +)] in Region 1 – 12. [file 12915_2024_1953_MOESM33_ESM.pdf]

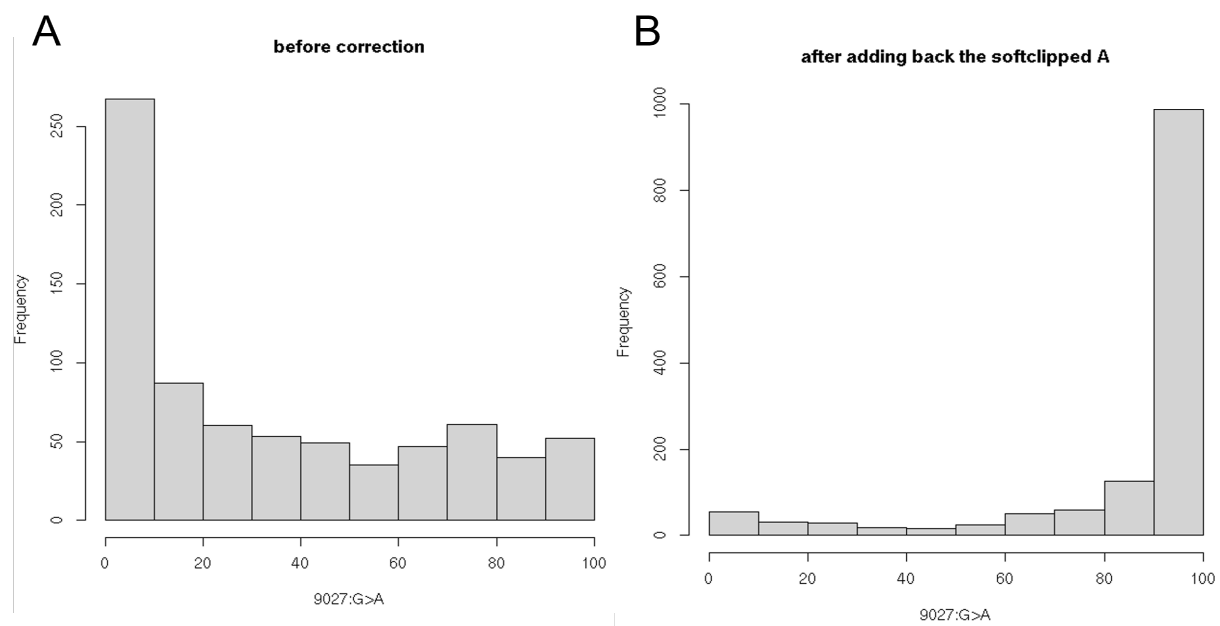

**Figure S22. Correction for Soft-clipping by STAR on 9027:G>A.**

Supplement: Supplementary file 35 — Additional file 35: Figure S22. Correction for Soft-clipping by STAR on 9027:G > A. The VAF difference between before and after patching the non-reference base As that were soft-clipped by STAR. (A) Before the correction. (B) After the correction. [file 12915_2024_1953_MOESM35_ESM.pdf]

A

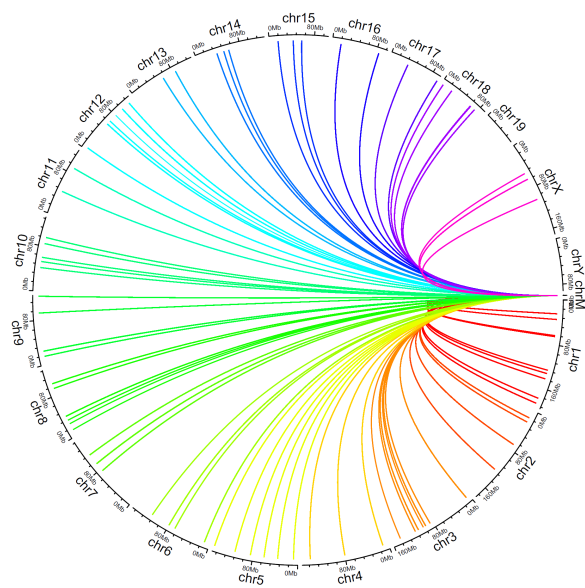

B

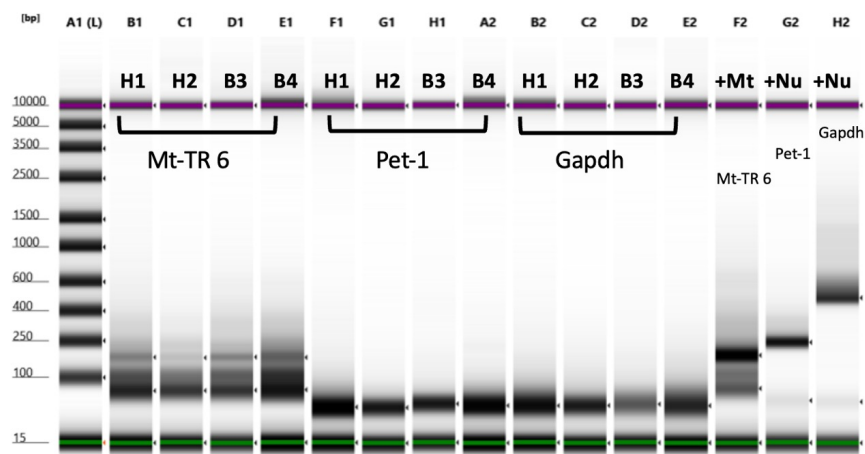

Figure S23. Mouse NUMTs Analysis on the SMITO dataset.

Supplement: Supplementary file 36 — Additional file 36: Figure S23. Mouse NUMTs Analysis on the SMITO dataset. (A) Homologous mtDNA shown as links; colors denote individual chromosomes. (B) Representative example of D5000 Agilent ScreenTape with PCR product from targeting single mt RCA product for a mt region (SNV6) and 2 independent nuclear regions (Pet-1 and Gapdh). [file 12915_2024_1953_MOESM36_ESM.pdf]

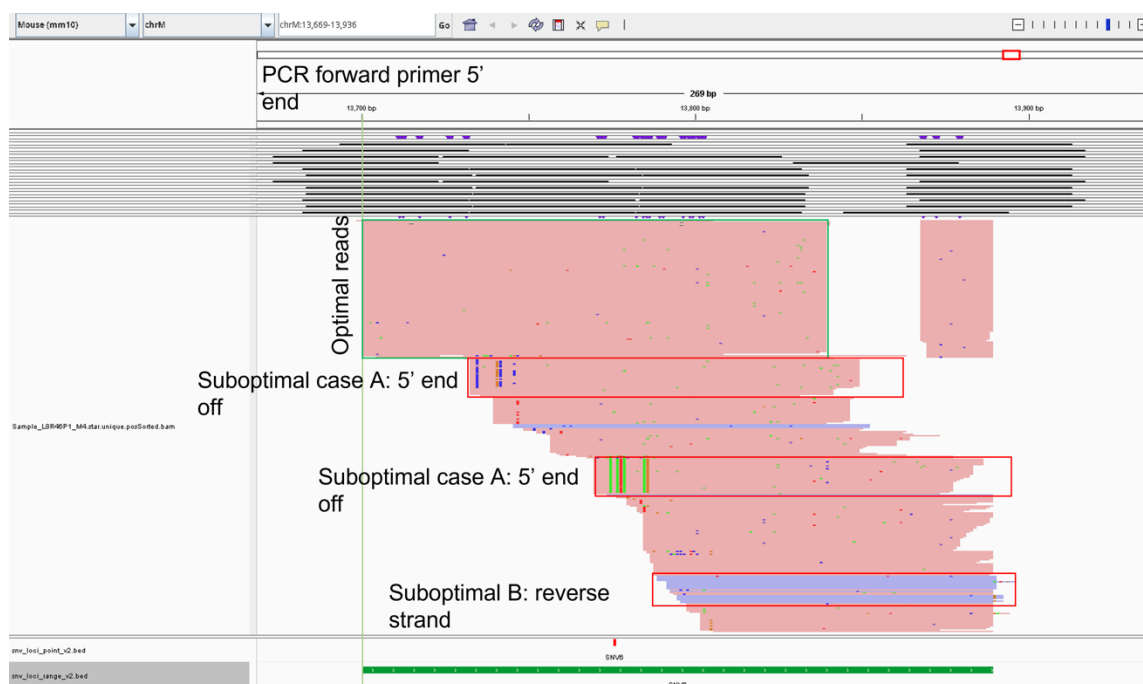

**Figure S25. Examples of suboptimal alignments and the mutations in linkage.**

Supplement: Supplementary file 39 — Additional file 39: Figure S25. Examples of suboptimal alignments and the mutations in linkage. The green box at the top highlights the optimal alignments, while the three red boxes represent suboptimal cases. [file 12915_2024_1953_MOESM39_ESM.pdf]

**A**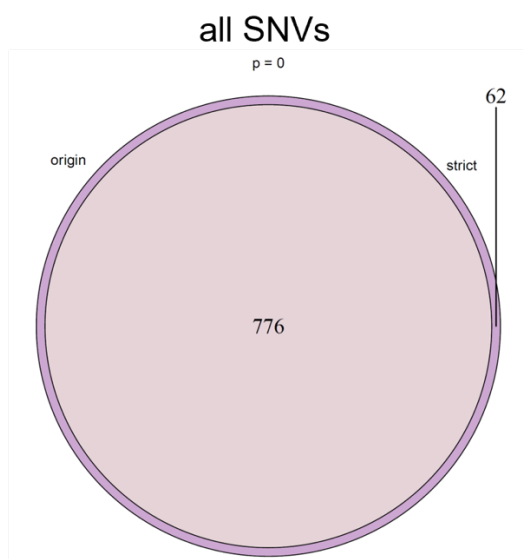**B**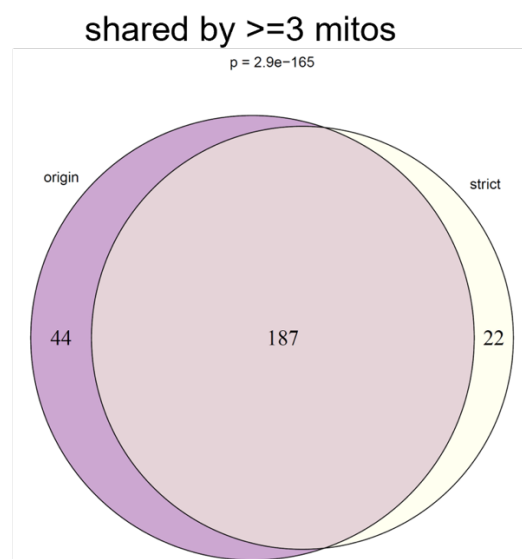

**Figure S26. Comparison between the permissive and the stricter filtering.**

Supplement: Supplementary file 40 — Additional file 40: Figure S26. Comparison between the permissive and the stricter filtering. (A) Venn diagram showing the overlap of all SNV sites between the permissive and the stricter filtering. (B) Venn diagram showing the overlap of the SNV sites shared by at least 3 mitochondria between the permissive and the stricter filtering. [file 12915_2024_1953_MOESM40_ESM.pdf]
