# Supplementary material for: Single-mitochondrion sequencing uncovers distinct mutational patterns and heteroplasmy landscape in mouse astrocytes and neurons
Source: BMC Biol. 2024 Jul 29;22:162. doi: 10.1186/s12915-024-01953-7 (PMC11287894; doi:10.1186/s12915-024-01953-7)
Supplement: Supplementary file 6 — Additional file 6: Figure S4. Comparison of Cells and Mitochondria Sharing Deleterious and Tolerated Nonsynonymous SNVs. (A) Number of cells or (B) mitochondria sharing the nonsynonymous SNVs, which were categorized as “deleterious” and “tolerated” by SIFT (for high-confidence only). The p-value is from Wilcoxon’s rank-sum test. [file 12915_2024_1953_MOESM6_ESM.pdf]

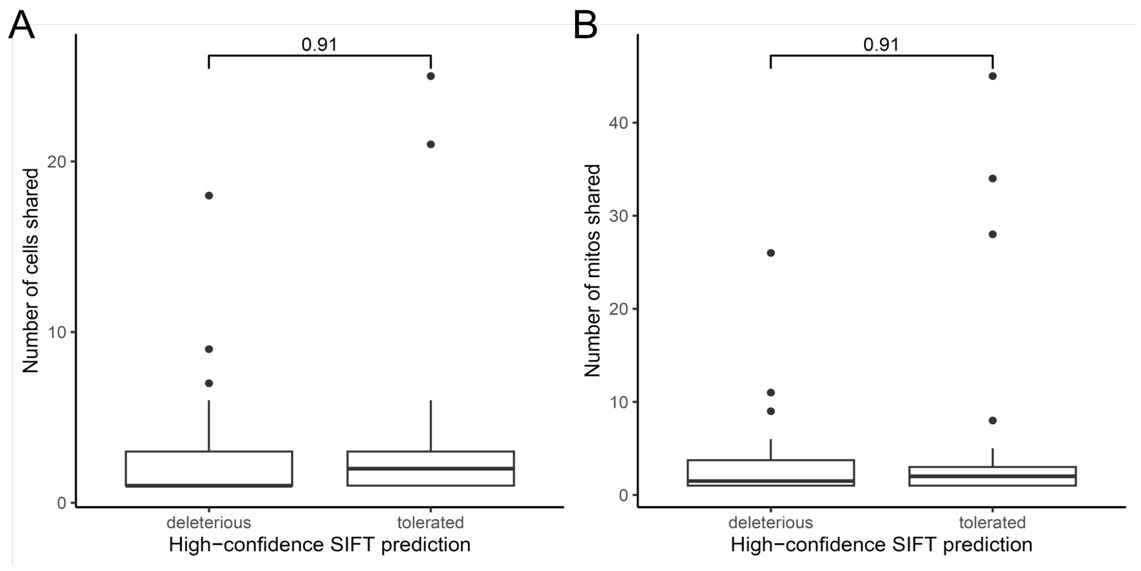

Figure S4. Comparison of Cells and Mitochondria Sharing Deleterious and Tolerated Nonsynonymous SNVs.
