# Supplementary material for: Single-mitochondrion sequencing uncovers distinct mutational patterns and heteroplasmy landscape in mouse astrocytes and neurons
Source: BMC Biol. 2024 Jul 29;22:162. doi: 10.1186/s12915-024-01953-7 (PMC11287894; doi:10.1186/s12915-024-01953-7)
Supplement: Supplementary file 7 — Additional file 7: Figure S5. Illustration of the anti-codon base changes in a tRNA SNV 9423:C > T. [file 12915_2024_1953_MOESM7_ESM.pdf]

The Ac-loop of mt-Tg (5' -> 3')

-2-101 23

CT[**TCC**]AA becomes

CT[**TCT**]AA

Figure S5. Illustration of the anti-codon base changes in a tRNA SNV 9423:C>T.
