# Supplementary material for: Single-mitochondrion sequencing uncovers distinct mutational patterns and heteroplasmy landscape in mouse astrocytes and neurons
Source: BMC Biol. 2024 Jul 29;22:162. doi: 10.1186/s12915-024-01953-7 (PMC11287894; doi:10.1186/s12915-024-01953-7)
Supplement: Supplementary file 11 — Additional file 11: Table S1. List of the genome accession numbers of the mouse strains, species and populations. [file 12915_2024_1953_MOESM11_ESM.docx]

**Table S1: List of the genome accession numbers of the mouse strains (A) , species (B) and populations (C-D).**

**Table S1A**

| Strain | Accession |
| --- | --- |
| 129S1/SvImJ | GCA_001624185.1 |
| A/J | GCA_001624215.1 |
| AKR/J | GCA_001624295.1 |
| BALB/cJ | GCA_001632525.1 |
| C3H/HeJ | GCA_001632575.1 |
| C57BL/6NJ | GCA_001632555.1 |
| CAST/EiJ | GCA_001624445.1 |
| CBA/J | GCA_001624475.1 |
| DBA/2J | GCA_001624505.1 |
| FVB/NJ | GCA_001624535.1 |
| LP/J | GCA_001632615.1 |
| NOD/ShiLtJ | GCA_001624675.1 |
| NZO/HlLtJ | GCA_001624745.1 |
| PWK/PhJ | GCA_001624775.1 |
| SPRET/EiJ | GCA_001624865.1 |
| WSB/EiJ | GCA_001624835.1 |

**Table S1B**

| Species | Accession |
| --- | --- |
| Mus caroli | NC_025268.1 |
| Mus cervicolor | KJ530560.1 |
| Mus cookii | NC_025270.1 |
| Mus famulus | NC_030342.1 |
| Mus fragilicauda | NC_025287.1 |
| Mus spretus | CM004188.1 |
| Mus terricolor | NC_010650.1 |

**Table S1C**

| Population (castaneus) | Accession |
| --- | --- |
| TW3 | gi\|574587513\|gb\|KF781664.1\| |
| CAST/EiJ | gi\|1022526666\|gb\|CM004181.1\| |
| PX | gi\|574587457\|gb\|KF781660.1\| |
| TW2 | gi\|574587499\|gb\|KF781663.1\| |
| GX | gi\|574587471\|gb\|KF781661.1\| |
| WH | gi\|574587429\|gb\|KF781658.1\| |
| GZ | gi\|574587485\|gb\|KF781662.1\| |
| TW1 | gi\|574587415\|gb\|KF781657.1\| |
| KM | gi\|574587443\|gb\|KF781659.1\| |

**Table S1D**

| Population (domesticus) | Accession |
| --- | --- |
| Ker_Gui05 | gi\|422034567\|gb\|JX945978.1\| |
| Ker_PJDA0908 | gi\|422034553\|gb\|JX945977.1\| |
| Ker_PJDA0907 | gi\|422034539\|gb\|JX945976.1\| |
| Ker_PJDA0901 | gi\|422034525\|gb\|JX945975.1\| |
| Ker_PJDA03 | gi\|422034511\|gb\|JX945974.1\| |
| Ker_PJDA01 | gi\|422034497\|gb\|JX945973.1\| |
| Ker_PAF0939 | gi\|422034483\|gb\|JX945972.1\| |
| Ker_PAF0933 | gi\|422034469\|gb\|JX945971.1\| |
| Ker_Mayes46 | gi\|422034455\|gb\|JX945970.1\| |
| Ker_Mayes34 | gi\|422034441\|gb\|JX945969.1\| |
| Ker_Mayes19 | gi\|422034427\|gb\|JX945968.1\| |
| Ker_Jack0922 | gi\|422034413\|gb\|JX945967.1\| |
| Ker_Jack0912 | gi\|422034399\|gb\|JX945966.1\| |
| Ker_IB06 | gi\|422034385\|gb\|JX945965.1\| |
| Ker_Couv0903 | gi\|422034371\|gb\|JX945964.1\| |
| LE_4221 | gi\|482677759\|gb\|KC663618.1\| |
| Crl:CD1(ICR) | gi\|482677815\|gb\|KC663622.1\| |
| Ker_Jack0902 | gi\|422034581\|gb\|JX945979.1\| |
| BG_4235 | gi\|482677773\|gb\|KC663619.1\| |
| HB_4242 | gi\|482677787\|gb\|KC663620.1\| |
