# Supplementary material for: Single-mitochondrion sequencing uncovers distinct mutational patterns and heteroplasmy landscape in mouse astrocytes and neurons
Source: BMC Biol. 2024 Jul 29;22:162. doi: 10.1186/s12915-024-01953-7 (PMC11287894; doi:10.1186/s12915-024-01953-7)
Supplement: Supplementary file 16 — Additional file 16: Figure S10. Uniform Manifold Approximation and Projection (UMAP) graph across all single-mitochondria samples. Left, UMAP embedding of the single mitochondria color-coded by the mouse. Right, UMAP embedding of the single mitochondria color-coded by the cell. [file 12915_2024_1953_MOESM16_ESM.pdf]

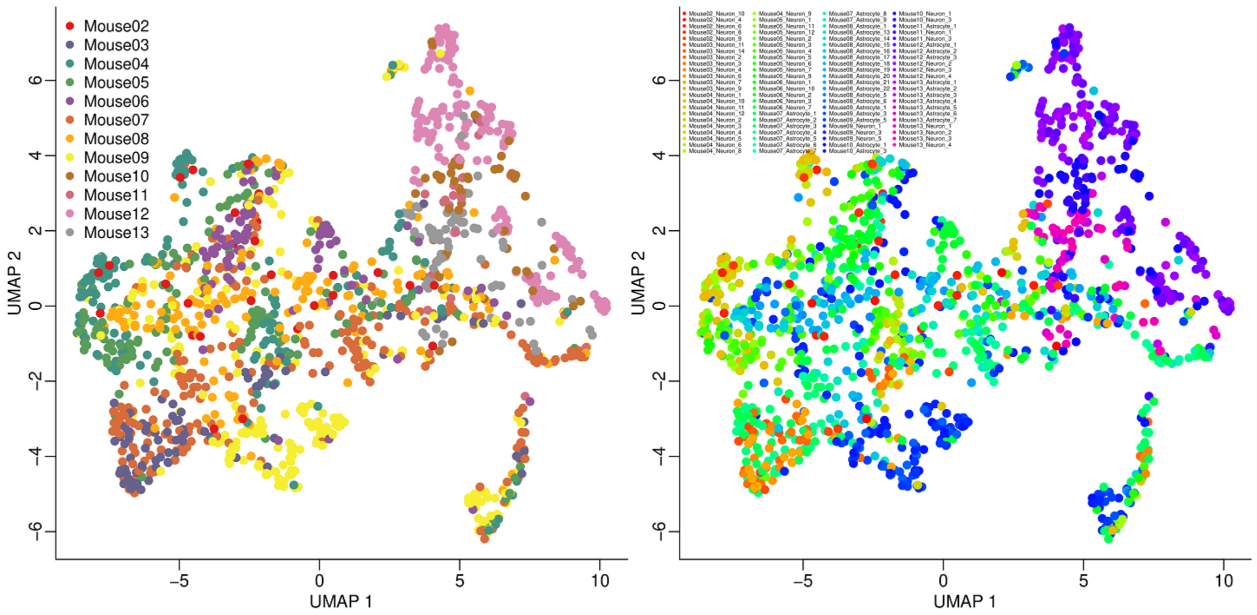

Figure S10. Uniform Manifold Approximation and Projection (UMAP) graph across all single-mitochondria samples.
