# Supplementary material for: Single-mitochondrion sequencing uncovers distinct mutational patterns and heteroplasmy landscape in mouse astrocytes and neurons
Source: BMC Biol. 2024 Jul 29;22:162. doi: 10.1186/s12915-024-01953-7 (PMC11287894; doi:10.1186/s12915-024-01953-7)
Supplement: Supplementary file 23 — Additional file 23: Figure S15. Single mitochondrion isolation from individual neuron or astrocyte cells based on Poisson statistics. (A) Flow cytometry characterization of antibody coated microbeads. (B) Gating strategy for isolating single mitochondrion from cell lysate of individual neuron or astrocyte staining by MitoTracker Red. Red dots indicated microbeads-only control, blue dots indicate microbeads captured unstained mitochondria as negative control. (C) MitoTracker gating on microbeads incubated with mitochondria stained by MitoTracker Red as positive control. (D) Comparing the MitoTracker signal from samples in B and C. Red plot indicate microbeads captured MitoTracker stained mitochondria. The orange plot depicts microbeads-only control. Blue plot depicts microbeads captured unstained mitochondria. (E) Experimental and theoretical analysis on single mt capture based on Poisson statistics. x-axis is the ratio of input mitochondria number to input microbead number. Y-axis is the probability of microbeads that captured a mitochondrion. Error bars indicate standard deviation from n = 3 independent experiments. [file 12915_2024_1953_MOESM23_ESM.pdf]

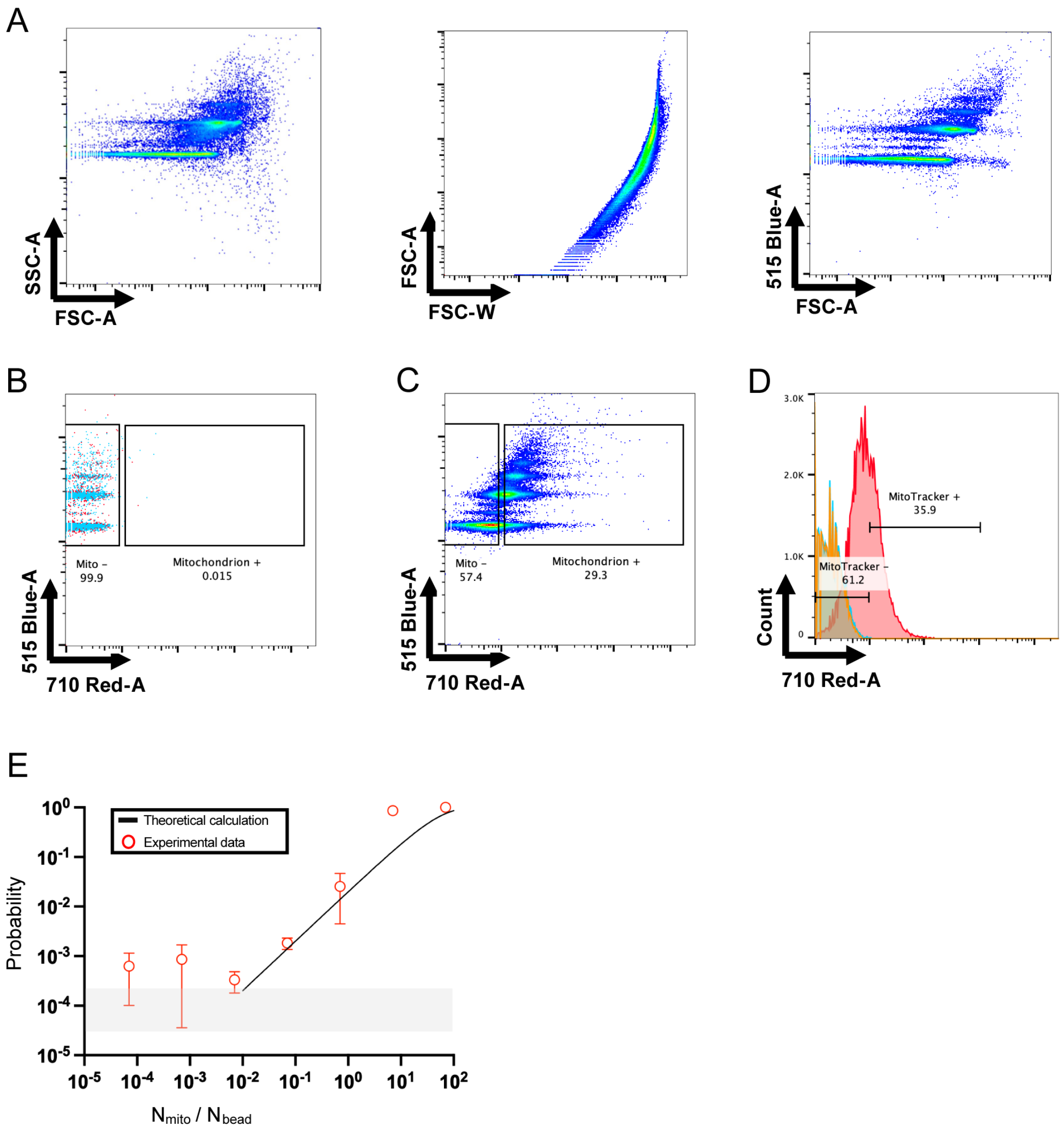


**Figure S15. Single mitochondrion isolation from individual neuron or astrocyte cells based on Poisson statistics.**
