# Supplementary material for: Single-mitochondrion sequencing uncovers distinct mutational patterns and heteroplasmy landscape in mouse astrocytes and neurons
Source: BMC Biol. 2024 Jul 29;22:162. doi: 10.1186/s12915-024-01953-7 (PMC11287894; doi:10.1186/s12915-024-01953-7)
Supplement: Supplementary file 25 — Additional file 25: Table S2. List of the sequences of the 10 barcodes (M1-10) for mitochondria multiplexing. [file 12915_2024_1953_MOESM25_ESM.docx]

**Table S2**. **List of the sequences of the 10 barcodes (M1-10) for mitochondria multiplexing.**

| ID | Spacer | Barcode | Full sequence |
| --- | --- | --- | --- |
| M1 | CGATT | ATCACG | CGATTATCACG |
| M2 | CGTAT | CGATGT | CGTATCGATGT |
| M3 | GCTAA | TTAGGC | GCTAATTAGGC |
| M4 | GCTTA | TGACCA | GCTTATGACCA |
| M5 | CGATT | ACAGTG | CGATTACAGTG |
| M6 | CGTAT | GCCAAT | CGTATGCCAAT |
| M7 | CGTAT | CAGATC | CGTATCAGATC |
| M8 | CGTAT | GATCAG | CGTATGATCAG |
| M9 | CGTAT | AGTCAA | CGTATAGTCAA |
| M10 | CGTAT | ACTGAT | CGTATACTGAT |
