# Supplementary material for: Single-mitochondrion sequencing uncovers distinct mutational patterns and heteroplasmy landscape in mouse astrocytes and neurons
Source: BMC Biol. 2024 Jul 29;22:162. doi: 10.1186/s12915-024-01953-7 (PMC11287894; doi:10.1186/s12915-024-01953-7)
Supplement: Supplementary file 26 — Additional file 26:Table S3. List of the sequences of the forward/reverse PCR primers for selected specific target regions. [file 12915_2024_1953_MOESM26_ESM.docx]

**Table S3.** List of the sequences of the forward/reverse PCR primers for selected specific target regions.

| Target region | SNV position | Gene | Forward primer sequence 5’ to 3’ | Reverse primer sequence 5’ to 3’ |
| --- | --- | --- | --- | --- |
| 1 | 9461 | mt-Nd3 | CTGACTTTTCCTATACGTCTCCA | GGGGGAGTCAGAATGCAACTA |
| 2 | 12913 | mt-Nd5 | CATAGCCTGGCAGACGAACA | ATTAGTAGGGCTCAGGCGTTG |
| 3 | 9027 | mt-Co3 | TGCAGGATTCTTCTGAGCGT | GGGCTTGATTTATGTGGTTTCGT |
| 4 | 6543 | mt-Co1 | CATCCCTTGACATCGTGCTTC | AATATGATGGCGAAGTGGGCT |
| 5 | 3816 | mt-Tq | AGAGGTTCAAGCCCTCTTATTT | CAACGTTTTCGGGGTATGGG |
| 6 | 13776 | mt-Nd6 | ACCAATCTCCCAAACCATCAAG | GGGGGATGTTGGTTGTGTTT |
| 7 | 3079 | mt-Nd1 | GCACCTACCCTATCACTCACAC | CGGCTCGTAAAGCTCCGAA |
| 8 | 16029 | D-Loop | GTCCGCAAAACCCAATCACC | TGATCAGGACATAGGGTTTGATAGT |
| 9 | 7612 | mt-Co2 | AGGCCGACTAAATCAAGCAA | AGGTTAACGCTCTTAGCTTC |
| 10 | 2651 | mt-Rnr2 | ACCTTACAAATAAGCGCTCTCAAC | TAGAATGGGGACGAGGAGTGT |
| 11 | 15191 | mt-Cytb | AATTGGGGGCCAACCAGTAG | TTCAGGTTTACAAGACCAGAGT |
| 12 | 1317 | mt-Rnr2 | ATAGAACTAGTACCGCAAGGGA | GTAGCTCGTTTGGTTTCGGG |
