# Supplementary material for: Single-mitochondrion sequencing uncovers distinct mutational patterns and heteroplasmy landscape in mouse astrocytes and neurons
Source: BMC Biol. 2024 Jul 29;22:162. doi: 10.1186/s12915-024-01953-7 (PMC11287894; doi:10.1186/s12915-024-01953-7)
Supplement: Supplementary file 27 — Additional file 27: Table S4. Edit distances between any pair of the mitochondrial-barcodes. [file 12915_2024_1953_MOESM27_ESM.docx]

**Table S4. Edit distances between any pair of the mitochondrial-barcodes.**

| Levenshtein | M1 | M2 | M3 | M4 | M5 | M6 | M7 | M8 | M9 | M10 |
| --- | --- | --- | --- | --- | --- | --- | --- | --- | --- | --- |
| M1 | 0 | 5 | 6 | 5 | 3 | 6 | 6 | 3 | 5 | 6 |
| M2 | 5 | 0 | 7 | 6 | 6 | 4 | 3 | 4 | 5 | 4 |
| M3 | 6 | 7 | 0 | 5 | 7 | 9 | 6 | 7 | 7 | 7 |
| M4 | 5 | 6 | 5 | 0 | 7 | 5 | 6 | 4 | 5 | 6 |
| M5 | 3 | 6 | 7 | 7 | 0 | 5 | 5 | 5 | 6 | 5 |
| M6 | 6 | 4 | 9 | 5 | 5 | 0 | 4 | 4 | 3 | 3 |
| M7 | 6 | 3 | 6 | 6 | 5 | 4 | 0 | 4 | 4 | 3 |
| M8 | 3 | 4 | 7 | 4 | 5 | 4 | 4 | 0 | 3 | 4 |
| M9 | 5 | 5 | 7 | 5 | 6 | 3 | 4 | 3 | 0 | 3 |
| M10 | 6 | 4 | 7 | 6 | 5 | 3 | 3 | 4 | 3 | 0 |
