# Supplementary material for: Single-mitochondrion sequencing uncovers distinct mutational patterns and heteroplasmy landscape in mouse astrocytes and neurons
Source: BMC Biol. 2024 Jul 29;22:162. doi: 10.1186/s12915-024-01953-7 (PMC11287894; doi:10.1186/s12915-024-01953-7)
Supplement: Supplementary file 38 — Additional file 38: Figure S24. Sequence comparison between UCSC GRCm38 (C57BL/6 J) and C57BL/6NJ from the Mouse Genomes Project in the region highlighting two differences. [file 12915_2024_1953_MOESM38_ESM.pdf]

```
chrM          ACTTCACCATCCTCCAAGCTTCAGAATACTTTGAAACATCATTCTCCATTTCAGATGGTA    9180
CM004277.1    ACTTCACCATCCTCCAAGCTTCAGAATACTTTGAAACATCATTCTCCATTTCAGATGGTA    9180
              ************************************************************

chrM          TCTATGGTTCTACATTCTTCATGGCTACTGGATTCCATGGACTCCATGTAATTATTGGAT    9240
CM004277.1    TCTATGGTTCTACATTCTTCATGGCTACTGGATTCCATGGACTCCATGTAATTATTGGAT    9240
              ************************************************************

chrM          CAACATTCCTTATTGTTTGCCTACTACGACAACTAAAATTTCACTTCACATCAAAACATC    9300
CM004277.1    CAACATTCCTTATTGTTTGCCTACTACGACAACTAAAATTTCACTTCACATCAAAACATC    9300
              ************************************************************

chrM          ACTTCGGATTTGAAGCCGCAGCATGATACTGACATTTTGTAGACGTAGTCTGACTTTTCC    9360
CM004277.1    ACTTCGGATTTGAAGCCGCAGCATGATACTGACATTTTGTAGACGTAGTCTGACTTTTCC    9360
              ************************************************************

chrM          TATACGTCTCCATTTATTGATGAGGATCTTACTCCCTTAGTATAATTAATATAACTGACT    9420
CM004277.1    TATACGTCTCCATTTATTGATGAGGATCTTACTCCCTTAGTATAATTAATATAACTGACT    9420
              ************************************************************

chrM          TCCAATTAGTAGATTCTGAATAAACCCAGAAGAGAGTAATTAACCTGTACACTGTTATCT    9480
CM004277.1    TCCAATTAGTAGATTCTGAATAAACCCAGAAGAGAGTAATCAACCTGTACACTGTTATCT    9480
              **************************************** *******************
      UCSC C57BL/6J
chrM          TCATTAATATTTTATTATCCCTAACGCTAATTCTAGTTGCATTCTGACTCCCCCAAATAA    9540
CM004277.1    TCATTAATATTTTATTATCCCTAACGCTAATTCTAGTTGCATTCTGACTCCCCCAAATAA    9540
              ************************************************************

chrM          ATCTGTACTCAGAAAAAGCAAATCCATATGAATGCGGATTCGACCCTACAAGCTCTGCAC    9600
CM004277.1    ATCTGTACTCAGAAAAAGCAAATCCATATGAATGCGGATTCGACCCTACAAGCTCTGCAC    9600
              ************************************************************
            Mouse Genomes Project C57BL/6NJ
chrM          GTCTACCATTCTCAATAAAATTTTTCTTGGTAGCAATTACATTTCTATTATTTGACCTAG    9660
CM004277.1    GTCTACCATTCTCAATAAAATTTTTCTTGGTAGCAATTACATTTCTATTATTTGACCTAG    9660
              ************************************************************

chrM          AAATTGCTCTTCTACTTCCACTACCATGAGCAATTCAAACAATTAAAACCTCTACTATAA    9720
CM004277.1    AAATTGCTCTTCTACTTCCACTACCATGAGCAATTCAAACAATTAAAACCTCTACTATAA    9720
              ************************************************************

chrM          TAATTATAGCCTTTATTCTAGTCACAATTCTATCTCTAGGCCTAGCATATGAATGAACAC    9780
CM004277.1    TAATTATAGCCTTTATTCTAGTCACAATTCTATCTCTAGGCCTAGCATATGAATGAACAC    9780
              ************************************************************

chrM          AAAAAGGATTAGAATGAACAGAGTAAATGGTAATTAGTTT-AAAAAAAATTAATGATTTC    9839
CM004277.1    AAAAAGGATTAGAATGAACAGAGTAAATGGTAATTAGTTTAAAAAAAAATTAATGATTTC    9840
              **************************************** *******************

chrM          GACTCATTAGATTATGATGATGTTCATAATTACCAATATGCCATCTACCTTCTTCAACCT    9899
CM004277.1    GACTCATTAGATTATGATGATGTTCATAATTACCAATATGCCATCTACCTTCTTCAACCT    9900
              ************************************************************

chrM          CACCATAGCCTTCTCACTATCACTTCTAGGGACACTTATATTTCGCTCTCACCTAATATC    9959
CM004277.1    CACCATAGCCTTCTCACTATCACTTCTAGGGACACTTATATTTCGCTCTCACCTAATATC    9960
              ************************************************************

chrM          CACATTACTATGCCTGGAAGGCATAGTATTATCCTTATTTATTATAACTTCAGTAACTTC    10019
CM004277.1    CACATTACTATGCCTGGAAGGCATAGTATTATCCTTATTTATTATAACTTCAGTAACTTC    10020
              ************************************************************

chrM          CCTAAACTCCAACTCCATAAGCTCCATACCAATCCCCATCACCATCTTAGTTTTCGCAGC    10079
CM004277.1    CCTAAACTCCAACTCCATAAGCTCCATACCAATCCCCATCACCATCTTAGTTTTCGCAGC    10080
```

Figure S24. Sequence comparison between UCSC GRCm38 (C57BL/6J) and C57BL/6NJ from the Mouse Genomes Project in the region highlighting two differences.
